# Global patterns and trends in ischemic stroke burden attributable to particulate matter pollution: changes from 1990 to 2021 and projections from 2022 to 2050

**DOI:** 10.3389/fpubh.2025.1599541

**Published:** 2025-06-26

**Authors:** Erman Wu, Riqing Su, Tong Tang, Jiakun Li, Maimaitili Mijiti, Yandong Li, Gaocai Zhang, Minghao Lian, Yongtao Zhang, Guohua Zhu, Dangmurenjiafu Geng

**Affiliations:** ^1^Neurosurgery Center of the First Affiliated Hospital of Xinjiang Medical University, Ürümqi, Xinjiang, China; ^2^Department of Computer Science and Information Technologies, Elviña Campus, University of A Coruña, A Coruña, Spain; ^3^Department of Urology, West China Hospital, Sichuan University, Chengdu, China

**Keywords:** global burden, ischemic stroke, air pollution, spatiotemporal trends, future projections

## Abstract

**Background:**

Stroke was the third leading cause of global deaths in 2021, linked to air pollution, especially particulate matter (PMP). Research shows that ischemic strokes are more affected by air pollution than hemorrhagic strokes. This study aims to evaluate the disease burden, trends, and future projections of ischemic stroke associated with PMP using the latest data.

**Methods:**

We used data from the 2021 Global Burden of Disease study to analyze the burden of ischemic stroke attributable to PMP from 1990 to 2021. Joinpoint regression was used to assess the trends (Average Annual Percentage Change, AAPC). Meanwhile, the Bayesian Age – Period – Cohort modeling method was used to project the burdens until 2050.

**Results:**

Globally, PMP-related ischemic stroke caused 905,600 deaths and 18.3 million DALYs in 2021, the highest levels in the past three decades PMP-related ischemic stroke deaths increased by 32.94% (1990–2021), yet ASDR declined by 46.65% (AAPC: −2.09, 95% CI: −2.45 to −1.72). Ambient particulate matter pollution (APMP) accounted for 66.6% of the burden in 2021 (vs. 45.9% in 1990), disproportionately affecting middle- and high-SDI regions. Conversely, household air pollution (HAP)-related burden declined but remained concentrated in low-SDI regions (80–82.5% in 2021). East Asia, South Asia, and Southeast Asia bore the highest absolute burdens, while Western Europe achieved the steepest ASR declines (AAPC for deaths: −6.55%). Projections to 2050 indicate rising ASRs. There was a negative correlation between SDI and ASRs, with APMP rising in middle-SDI nations and HAP persisting in low-SDI areas. Significant gender differences exist in the disease burden of PMP – induced ischemic stroke. Males generally have higher mortality rates and DALYs than females across most age groups, and the peak male mortality has been delayed over the past 30 years.

**Conclusion:**

This global analysis underscores the urgent need for targeted pollution control strategies to address the dual burden of ischemic stroke driven by APMP in high- and middle-income regions and HAP in low-resource settings, emphasizing the critical role of tailored interventions to mitigate health disparities and achieve sustainable development goals.

## 1 Introduction

Stroke was the third leading cause of global mortality in 2021, resulting in approximately 7.14 million deaths worldwide (1). When considering disability-adjusted life years (DALYs), stroke ranked fourth, accounting for around 160.4 million DALYs in 2021 (2). Ischemic stroke, which makes up 60–70% of all stroke cases, was responsible for an estimated 3.59 million deaths globally in 2021, marking a 55% increase from 1990. Concurrently, the DALYs attributed to ischemic stroke increased by 223%, surpassing 70.4 million in 2021 (3). This substantial rise underscores the growing global burden of ischemic stroke and underscores the need for targeted intervention strategies.

Risk factors for stroke can be categorized into non-modifiable and modifiable factors. It is estimated that nearly 90% of strokes are linked to modifiable risk factors, making their reduction a crucial strategy for preventing ischemic stroke (4). Extensive research has demonstrated a strong correlation between exposure to air pollution, regardless of duration, and the risk of stroke (5, 6). The evidence indicates that ischemic strokes are more significantly affected by air pollution compared to hemorrhagic strokes (7, 8). In recent years, the rapid industrialization and urbanization have exacerbated air pollution. The adverse effects of fine particulate matter (PM_2.5_) on human health have become a global concern. The detrimental impacts of PM_2.5_ on human health are mainly mediated through the following mechanisms: induction of oxidative stress, cytokine release, deoxyribonucleic acid (DNA) damage, altered gene expression, immune toxicity, inflammatory responses, and apoptosis (9).

As a prevalent form of air pollution, particulate matter pollution (PMP) is divided into ambient particulate matter pollution (APMP) and household air pollution (HAP). Globally, approximately 92% of the population lives in areas where PM_2.5_ concentrations exceed the WHO’s air quality guideline of 10 μg/m^3^ (10). In 2019, environmental PMP was the fourth leading contributor to the global stroke burden, accounting for 20.2% of the total burden (11). According to the GBD 2021 Risk Factors Collaborators, PMP was the leading contributor to the global disease burden in 2021, responsible for 8.0% of total DALYs (12). While HAP burden has decreased significantly, it remains a major risk, particularly in sub-Saharan Africa and South Asia (13).

Given PMP’s status as the top contributor to global disease burden in 2021, there is an urgent need to use the latest GBD 2021 data to assess the effects, trends, and future projections of ischemic stroke and its disease burden across different countries, regions, genders, and age groups. Liu et al. (8) conducted a study on the association between particulate matter pollution and stroke based on 2019 data, employing the Estimated Annual Percent Change (EAPC) instead of the Average Annual Percent Change (AAPC), The EAPC concisely quantifies long-term trends through a single percentage value, combining statistical robustness with cross-population comparability, making it an ideal tool for assessing linear trends. For analyzing the association between particulate matter pollution and ischemic stroke, the AAPC integrates heterogeneous fluctuations and multi-phase intervention impacts, more accurately capturing the complex effects of pollution exposure. When temporal trends exhibit non-linearity (e.g., phased implementation of pollution policies or pronounced seasonal variations), the AAPC provides a global annual average by weighting segmental change rates, rendering it more suitable than the EAPC for such environmental health studies (8).

Consequently, this study aims to: (1) evaluate the global disease burden (mortality and DALYs) of ischemic stroke attributable to particulate matter pollution (both APMP and HAP) using the latest GBD 2021 data; (2) analyze temporal trends from 1990 to 2021 utilizing the AAPC to robustly capture complex patterns; (3) project the future burden of PMP-attributable ischemic stroke up to 2035; and (4) characterize these burdens and trends across countries, regions, genders, and age groups. By providing this comprehensive, up-to-date, and methodologically refined analysis, our study fills a significant knowledge gap and offers crucial evidence to inform global and national strategies for mitigating the impact of air pollution on ischemic stroke.

## 2 Methods

### 2.1 Data sources

In 2024, the GBD introduced the updated GBD 2021 database (2). This research presents findings on the incidence, prevalence, mortality, DALYs, years lived with disability (YLDs), and years of life lost (YLL) for 371 diseases across 204 countries and regions, incorporating data on 88 risk factors from 1990 to 2021, along with the associated uncertainty intervals (UIs). Detailed descriptions of the original data and the methodology employed in the GBD 2021 study have been published previously (1, 2, 12). Data on the impact of PMP on ischemic stroke were retrieved from the Global Health Data Exchange (GHDE) using the GBD Results Tool^1^. In this study, we compiled data on the number and age-standardized rates of deaths, DALYs, YLDs, and YLLs for ischemic stroke related to PMP from 1990 to 2021, including their 95% UIs. We also collected sex, age, the Socio-Demographic Index (SDI), and geographic location information from GHDE to perform a more thorough analysis. Additionally, we obtained rates of deaths, YLDs, DALYs, and YLLs related to ischemic stroke caused by PMP, categorized by age group.

### 2.2 Definition

In the GBD 2021, PMP is categorized into two types: APMP and HAP. APMP is defined as the population-weighted annual average mass concentration of particles with an aerodynamic diameter of less than 2.5 micrometers (PM_2.5_) per cubic meter of air. HAP is assessed based on factors such as the population using solid fuels in households and the concentration of particulate matter. Data on PMP were collected from both national and regional monitoring stations, as well as various environmental pollution databases (12). An ischemic stroke refers to a sudden episode of neurological impairment that results from an infarction, which is the death of tissue due to a lack of blood supply. According to the 10th revision of the International Classification of Diseases (ICD-10), ischemic stroke can be categorized under several codes, including G45–G46.8, I63–I63.9, I65–I66.9, I67.2–I67.848, and I69.3–I69.4 (14).

Age-standardized rates (ASRs) are calculated per 100,000 individuals, using a standard age distribution to allow fair comparisons between regions. DALYs serve as a measure of the disease burden, incorporating both the YLL and the YLD, with detailed definitions previously published (15). The SDI quantifies development by integrating income, education, and fertility metrics, categorizing regions into five stages: low (0–0.4658), low-middle (0.4658–0.6188), middle (0.6188–0.7120), high-middle (0.7120–0.8103), and high SDI (0.8103–1), mirroring population affluence and educational attainment. We utilized these pre-existing SDI values corresponding to the locations and years in our ischemic stroke burden dataset. The detailed SDI methodology is described in the GBD 2021 capstone publications and foundational SDI methodology papers (12, 16).

The SDI is a composite index of socio-demographic development status strongly correlated with health outcomes. It represents the mean education level for those aged 15 years or older, the geometric mean of 0 to 1 indices of the total fertility rate in those under 25 years old, and lag-distributed income per capita. It is crafted to assess and compare socio-economic development levels across various regions, countries, or populations. The 204 countries and territories are categorized into five SDI quintiles ranging from low to high and are also divided geographically into 21 GBD regions (17).

### 2.3 Statistical analysis

The study compared the death, DALYs, YLL and YLDs between the sexes, age, SDI (five categories), regions (21 GBD regions), and countries (204 countries and territories). The temporal trend was evaluated using the Join point Regression Program (Version 5.0.2), and the average annual percent change (AAPC) was calculated during 1990–2021 with default parameters. To investigate the factors influencing PAP, the association between ASRs and SDI, it was assessed at the national level using generalized linear model (GLM) and the Pearson test was used to determine statistical significance. Statistical analyses and the visualization of results were conducted using the R software (version 4.3.1, R Core Team).

### 2.4 Projection analysis

The projection analysis is designed to implement Bayesian age-period-cohort models, with a specific emphasis on projections. Bayesian age-period-cohort models (BAPC) employ integrated nested Laplace approximations (INLA) to facilitate comprehensive Bayesian inference (18). This study adopts the BAPC model due to its capability to effectively disentangle the independent effects of age, period, and cohort, aligning with the interaction mechanisms of historical exposure accumulation and demographic changes in environmental health issues. By integrating the INLA algorithm, the BAPC model efficiently handles complex random effects within the Bayesian framework and quantifies uncertainties in long-term projections. Compared to traditional models (e.g., ARIMA and Nordpred) or machine learning approaches, the BAPC model is more suitable for mechanism-driven policy simulations, balancing interpretability and extrapolation robustness (19, 20).

In our study, we implement a BAPC model, integrated with the INLA method, to decompose temporal trends into age, period, and cohort effects. This is achieved using fifth-order B-splines (gf = 5) and intrinsic Gaussian Markov Random Field priors, with a Poisson likelihood assumption and INLA for efficient posterior estimation. To ensure the model’s robustness, we conducted retrospective comparisons (1990–2021), performed Geweke diagnostics to assess convergence, and carried out sensitivity analyses using alternative priors. For the projection phase (2022–2050), we applied World Health Organization (WHO) population weights to standardize age structures. The BAPC model not only generates age-specific incidence and mortality rates but also produces age-standardized projected rates. This capability is particularly advantageous for adjusting differences in population age structures, thereby facilitating more accurate comparisons across time and diverse populations.

## 3 Results

### 3.1 Global disease burden of ischemic stroke attributable to PMP from 1990 to 2021 and projections from 2022 to 2025

**Figure 1:**
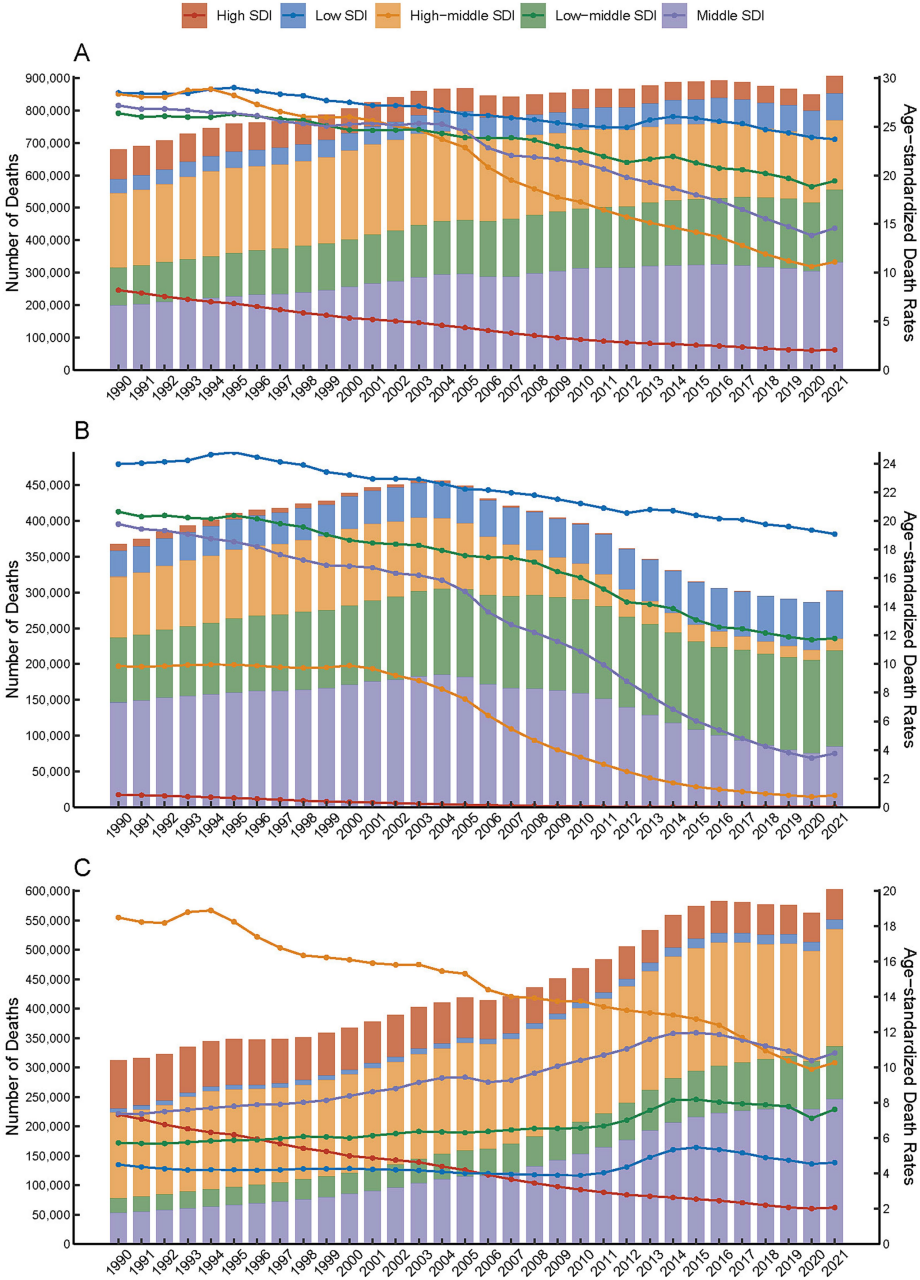
Global deaths from ischemic stroke attributable to particulate matter pollution, categorized by five levels of Socio-demographic Index (SDI), and presented by both number (bar plot) and age-standardized rate (line plot) from 1990 to 2021. **(A)** Total particulate matter pollution. **(B)** Household air pollution. **(C)** Ambient particulate matter pollution.

Globally, the number of deaths attributed to ischemic stroke related to total PMP rose by 32.94% from 1990 to 2021, increasing from 681,180 to 905,600 deaths. In contrast, the ASDR for ischemic stroke due to total PMP decreased by 46.65%, declining from 20.65 per 100,000 population in 1990 to 11.01 per 100,000 in 2021, with an AAPC of −2.09% (95% CI: −2.45 to −1.72) (Table 1). Similarly, the number of DALYs increased by 29.79%, rising from 14.1 million in 1990 to 18.3 million in 2021. Meanwhile, the age-standardized rates of DALYs, YLLs, and YLDs due to ischemic stroke attributable to PMP decreased by 21.70, 46.66, and 21.1%, respectively, with AAPCs of −1.94 (95% CI: −2.29 to −1.58), −2.05 (95% CI: −2.22 to −1.87), and −0.82 (95% CI: −0.93 to −0.71) (Figure 1A and Supplementary Tables S1–S3). It is particularly noteworthy that the global numbers of deaths, DALYs, YLLs, and YLDs associated with both APMP and HAP in 2021 were higher than past 5 years (Figure 1 and Supplementary Figures S1–S3).

**Table 1:** Number and age-standardized death rates of ischemic stroke attributable to particulate matter pollution, with temporal trends from 1990 to 2021.

Characteristics	1990		2021		1990–2021
	Number of deaths no. × 10^3^ (95% UI)	Age-standardized deaths rate per 100,000 (95% UI)	Number of deaths no. × 10^3^ (95% UI)	Age-standardized deaths rate per 100,000 (95% UI)	AAPC (95% CI)
Global	681.18 (537.1–836.7)	20.65 (16.22–25.54)	905.6 (694.79–1144.79)	11.01 (8.44–13.96)	−2.09 (−2.45–1.72)
Female	368.26 (290.15–464.4)	19.1 (14.95–24.08)	438 (341.91–558.39)	9.34 (7.3–11.91)	−2.4 (−2.63–−2.17)
Male	312.92 (243.36–385.2)	22.68 (17.65–28.14)	467.6 (352.73–593.16)	13.18 (9.97–16.74)	−1.78 (−2.04–−1.51)
Low SDI	42.97 (33.19–56.17)	28.47 (22.04–36.75)	83.16 (65.38–106.74)	23.7 (18.67–30.13)	−0.58 (−0.69–−0.48)
Low-middle SDI	115.54 (89.85–142.88)	26.37 (20.38–32.22)	222.69 (177.83–272.12)	19.43 (15.4–23.66)	−1.06 (−1.17–−0.95)
Middle SDI	199.47 (158.51–246.22)	27.17 (21.51–33.58)	331.72 (244.4–435.83)	14.57 (10.74–19.19)	−2.07 (−2.4–−1.73)
High-middle SDI	230.6 (167.49–300.33)	28.35 (20.42–37.17)	215.07 (159.89–283.03)	11.1 (8.24–14.6)	−3.01 (−3.44–−2.58)
High SDI	91.57 (61.27–129.88)	8.21 (5.47–11.67)	52.22 (37.75–68.85)	2.08 (1.53–2.74)	−4.35 (−4.59–−4.1)
Australasia	0.38 (0.01–1.12)	1.76 (0.06–5.18)	0.47 (0.27–0.72)	0.7 (0.4–1.07)	−2.78 (−3.52–−2.05)
Oceania	0.34 (0.24–0.46)	20.81 (14.95–27.5)	0.73 (0.52–1.02)	16.24 (11.5–22.33)	−0.8 (−0.85–−0.76)
East Asia	209.47 (163.69–264.57)	35.24 (27.65–43.96)	366.84 (265.78–483.72)	19.21 (13.98–25.3)	−1.99 (−2.39–−1.58)
Central Asia	8.16 (4.62–12.14)	20.23 (11.39–30.17)	9.56 (7.07–12.49)	15.02 (11.05–19.63)	−1.07 (−1.78–−0.35)
South Asia	84.03 (63.49–110.07)	21.2 (16.08–27.44)	184.32 (144.13–239.03)	15.84 (12.45–20.15)	−0.98 (−1.69–−0.26)
Southeast Asia	59.22 (45.81–71.9)	32.89 (25.52–39.91)	89.44 (60.31–122.86)	17.92 (12.17–24.51)	−2.02 (−2.25–−1.79)
High-income Asia Pacific	11.72 (3.25–23.82)	6.84 (1.86–14)	12.13 (7.02–18.32)	1.75 (1.03–2.6)	−4.33 (−4.7–−3.97)
Eastern Europe	84.08 (41.99–130.48)	34.91 (17.31–54.21)	29.05 (18.16–44.01)	8.03 (5.02–12.17)	−4.67 (−5.31–−4.02)
Central Europe	46.8 (27.53–65.65)	36.48 (21.47–51.21)	22.87 (16.88–32.69)	9.36 (6.91–13.38)	−4.36 (−4.63–−4.09)
Western Europe	56.67 (27.34–93.86)	9.34 (4.49–15.49)	14.89 (9.92–21.25)	1.15 (0.77–1.64)	−6.55 (−6.96–−6.14)
High-income North America	10.32 (3.96–18.35)	2.77 (1.06–4.92)	4.01 (1.98–6.73)	0.53 (0.26–0.89)	−5.14 (−5.65–−4.63)
Andean Latin America	2.33 (1.76–2.93)	13.55 (10.25–17.12)	2.05 (1.33–2.97)	3.71 (2.41–5.38)	−4.06 (−4.46–−3.67)
Central Latin America	6.62 (4.31–9.08)	10.27 (6.63–14.07)	6.09 (4.12–8.81)	2.65 (1.79–3.83)	−4.26 (−4.58–−3.94)
Southern Latin America	4.17 (2.26–6.36)	10.27 (5.53–15.67)	2.35 (1.43–3.55)	2.52 (1.54–3.82)	−4.48 (−4.77–−4.2)
Tropical Latin America	12.15 (7.25–18.17)	17.94 (10.72–26.6)	7.3 (4.24–11.25)	3.02 (1.76–4.66)	−5.63 (−5.87–−5.39)
Caribbean	3.07 (2.1–4.42)	13.38 (9.12–19.26)	4.34 (2.94–6)	7.99 (5.42–11.04)	−1.6 (−1.93–−1.27)
North Africa and Middle East	38.1 (29.03–47.95)	30.99 (23.59–38.83)	68.28 (51.43–83.57)	19.45 (14.79–24.12)	−1.57 (−1.7–−1.43)
Eastern Sub-Saharan Africa	12.37 (9.44–16.15)	25.91 (20.25–33.71)	25.18 (19.52–31.4)	22.61 (17.48–28.18)	−0.43 (−0.54–−0.32)
Central Sub-Saharan Africa	4.07 (2.96–5.37)	31.31 (23–40.91)	8.14 (5.52–11.78)	25.92 (17.42–37.82)	−0.61 (−0.75–−0.47)
Southern Sub-Saharan Africa	3.11 (2.21–4.05)	14.98 (10.6–19.67)	5.71 (4.14–7.51)	13.57 (9.77–18.02)	−0.26 (−0.72–0.19)
Western Sub-Saharan Africa	24 (17.68–31.89)	37.69 (27.64–49.78)	41.87 (31.96–53.45)	30.96 (23.68–39.26)	−0.63 (−0.75–−0.5)

**Figure 2:**
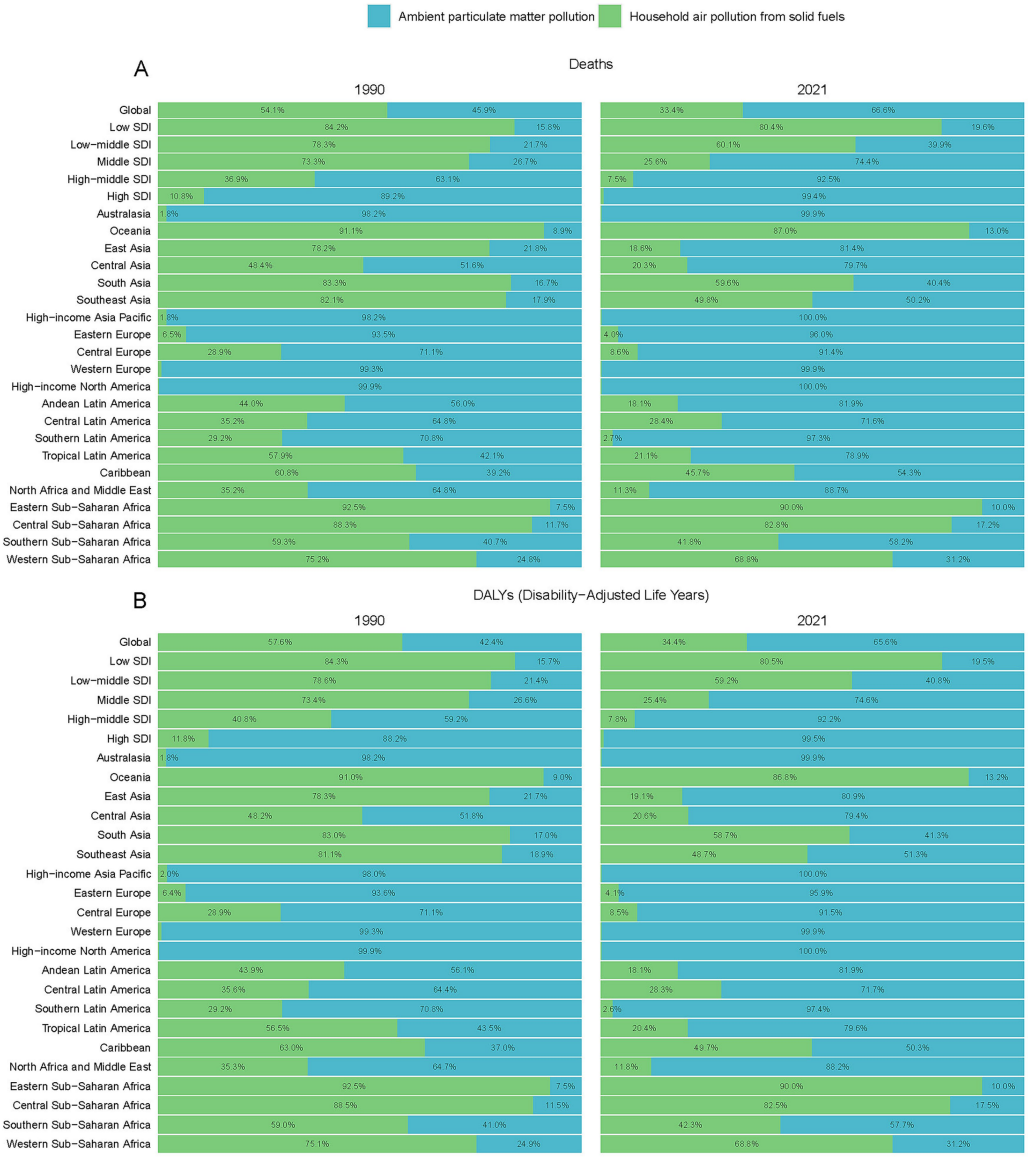
Contribution of mortality figures and percentage due to household air pollution and ambient particulate matter pollution, on a global scale and by region, from 1990 to 2021 **(A)** Death, **(B)** DALYs.

Furthermore, the global numbers of ischemic stroke deaths, DALYs, YLLs, and YLDs attributable to HAP increased from 1990, peaked around 2003–2004, and subsequently declined until 2021, with a slight rise thereafter (Figure 1B and Supplementary Figures S1B, S2B, S3B). In contrast, the global numbers associated with APMP peaked in 2016–2017, followed by a gradual decline until a slight increase in 2021 (Figure 1C and Supplementary Figures S1C, S2C, S3C). Additionally, compared to 1990, the global proportion of ischemic stroke burden (measured in terms of deaths and all-cause DALYs) attributable to APMP increased from 45.9 to 66.6% in 2021, whereas the contribution from HAP decreased from 54.1 to 33.4% (Figure 2 and Supplementary Figures S4–S6).

**Figure 3:**
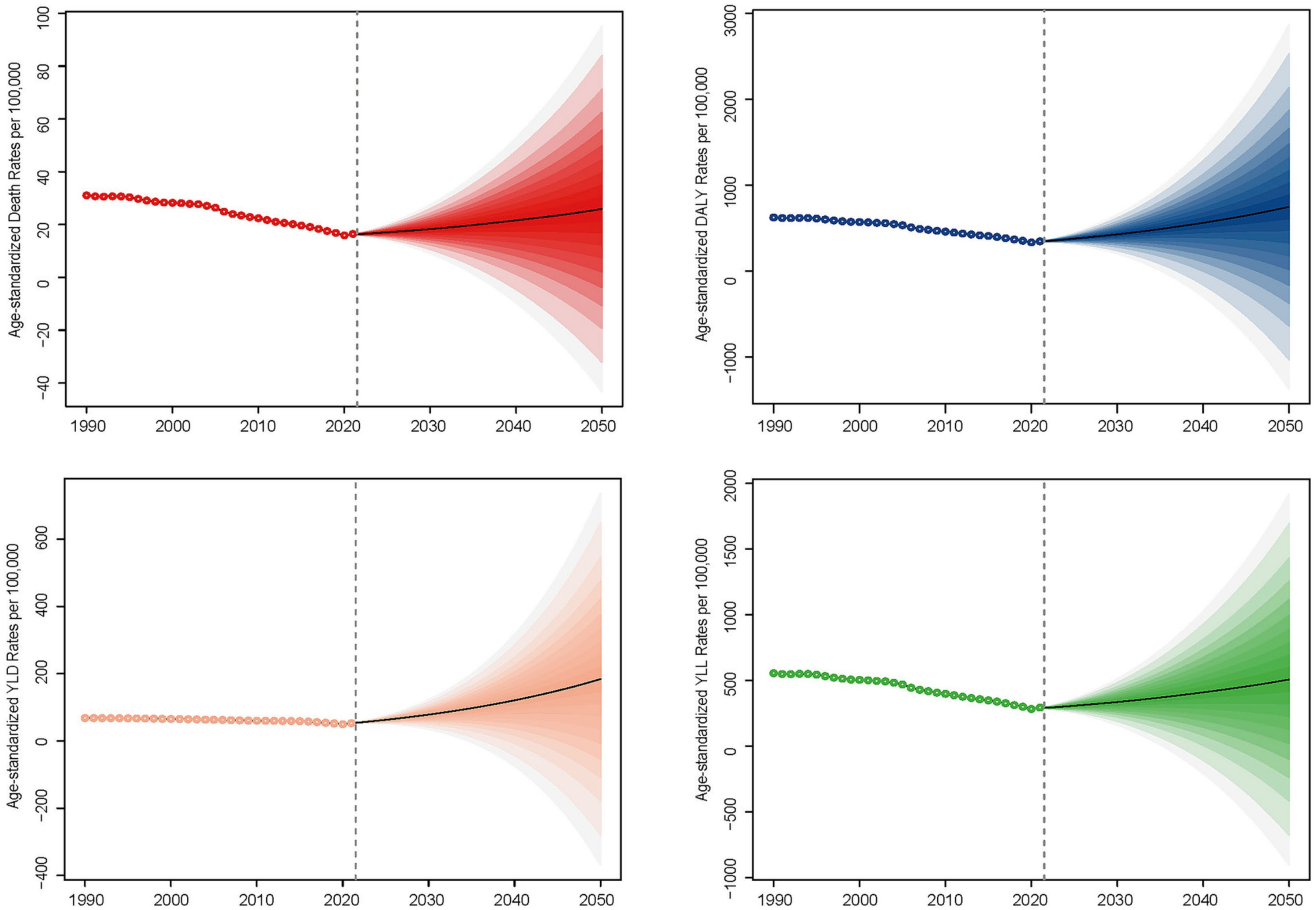
Global projections for the period from 2022 to 2050, indicating that the age-standardized rates of deaths, DALYs (Disability-Adjusted Life Years), YLLs (Years of Life Lost), and YLDs (Years Lived with Disability) attributable to ischemic stroke are associated with total particulate matter pollution.

Global projections for the period from 2022 to 2050 indicate that age-standardized rates of deaths, DALYs, YLLs, and YLDs attributable to ischemic stroke related to total PMP are expected to continue rising (Figure 3).

### 3.2 Global disease burden of ischemic stroke attributable to PMP from 1990 to 2021 by SDI

The burden of ischemic stroke attributed to total PMP reveals significant variations across different SDI regions regarding deaths, DALYs, YLLs, and YLDs. Among these, high-middle, low-middle, and middle SDI regions represent most of the disease burden (Figure 1A and Supplementary Figures S1A, S2A, S3A). In 1990, high-middle SDI regions reported the highest figures for deaths, DALYs, and YLLs. However, by 2021, middle SDI regions had overtaken high-middle SDI regions in all three metrics. Meanwhile, YLDs were consistently highest in middle SDI regions for both 1990 and 2021. Conversely, low SDI regions exhibited the least severe rates for these metrics in 1990, while high SDI regions showed the least severe rates in 2021 for deaths, DALYs, YLLs, and YLDs (Figures 2A, B; Supplementary Figures S4–S6; Table 1; Supplementary Tables S1–S3). From 1990 to 2021, the age-standardized rates of deaths, DALYs, YLLs, and YLDs associated with ischemic stroke attributable to PMP declined across all SDI subgroups, with the most substantial declines observed in high SDI countries. Specifically, the AAPC for deaths was −4.35 (95% CI: −4.59 to −4.10), for DALYs, it was −3.75 (95% CI: −4.00 to −3.50), for YLLs, it was −4.22 (95% CI: −4.51 to −3.94), with an AAPC for YLDs of −1.70 (95% CI: −1.77 to −1.63) (Table 1 and Supplementary Tables S1–S3).

The analysis of ischemic stroke attributable to HAP revealed a decreasing trend in age-standardized rates of deaths, DALYs, YLDs, and YLLs across all SDI subgroups. The highest rates were observed in low SDI regions, followed by low-middle, middle, high-middle, and eventually high SDI regions, which exhibited the lowest rates (Figure 1B and Supplementary Figures S1B, S2B, S3B). Conversely, when examining ischemic stroke linked to APMP, a different pattern emerged. The rates of deaths, DALYs, and YLLs decreased in high-middle and high SDI regions, while they increased in low-middle, middle, and low SDI regions (Figure 1C and Supplementary Figures S1C, S3C). Moreover, YLD rates for ischemic stroke due to APMP rose until peaking in 2015 and then declined by 2021 in low and high-middle SDI regions, whereas high SDI regions exhibited a continuous decline (Supplementary Figure S2C). By 2021, high and high-middle SDI regions shouldered the predominant burden of ischemic stroke attributable to APMP, accounting for 92 to 99.5% of the total PMP (Figure 2 and Supplementary Figures S4–S6). In contrast, low SDI regions primarily contended with the burden of HAP -related ischemic stroke, representing 80 to 82.5% of the total PMP in 2021 (Figure 2 and Supplementary Figures S4–S6).

### 3.3 Global disease burden of ischemic stroke attributable to PMP from 1990 to 2021 by GBD regions

Among the 21 GBD regions, East Asia, South Asia, and Southeast Asia were the top three regions with the highest number of deaths, DALYs, YLLs, and YLDs attributable to PMP in 2021. In East Asia, the primary contributor in 1990 was HAP, whereas in 2021, it shifted to APMP (Figure 2 and Supplementary Figures S4–S6).

High SDI regions, including Western Europe, Australasia, High-income Asia Pacific, and High-income North America, were primarily facing an ischemic stroke burden attributable to APMP, accounting for more than 98% of the total PMP in both 1990 and 2019 (Figure 2 and Supplementary Figures S4–S6). In High-Income North America, the ischemic stroke burden due to PMP was entirely caused by APMP in both years. Low SDI regions, including Sub-Saharan Africa and Oceania, were predominantly affected by HAP related to ischemic stroke during the same years (Figure 2 and Supplementary Figures S4–S6).

The age-standardized rates of ischemic stroke burden related to total PMP demonstrated a decreasing trend across the 21 GBD regions. Western Europe exhibited the most substantial decreases. The AAPC values for the total PMP were −6.55 (95% CI: −6.96 to −6.14) for deaths, −6.18 (95% CI: −6.51 to −5.85) for DALYs, −6.81 (95% CI: −7.15 to −6.47) for YLLs, and −3.43 (95% CI: −3.50 to −3.36) for YLDs (Table 1 and Supplementary Tables S1–S3). Conversely, the smallest decreases were observed in Southern and Eastern Sub-Saharan Africa. In Southern Sub-Saharan Africa, the AAPC values for the total PMP were −0.26 (95% CI: −0.72 to −0.19) for deaths and −0.32 (95% CI: −0.80 to −0.15) for YLLs. Eastern Sub-Saharan Africa recorded AAPC values for the total PMP of −0.45 (95% CI: −0.54 to −0.36) for DALYs and −0.27 (95% CI: −0.28 to −0.26) for YLDs.

**Figure 4:**
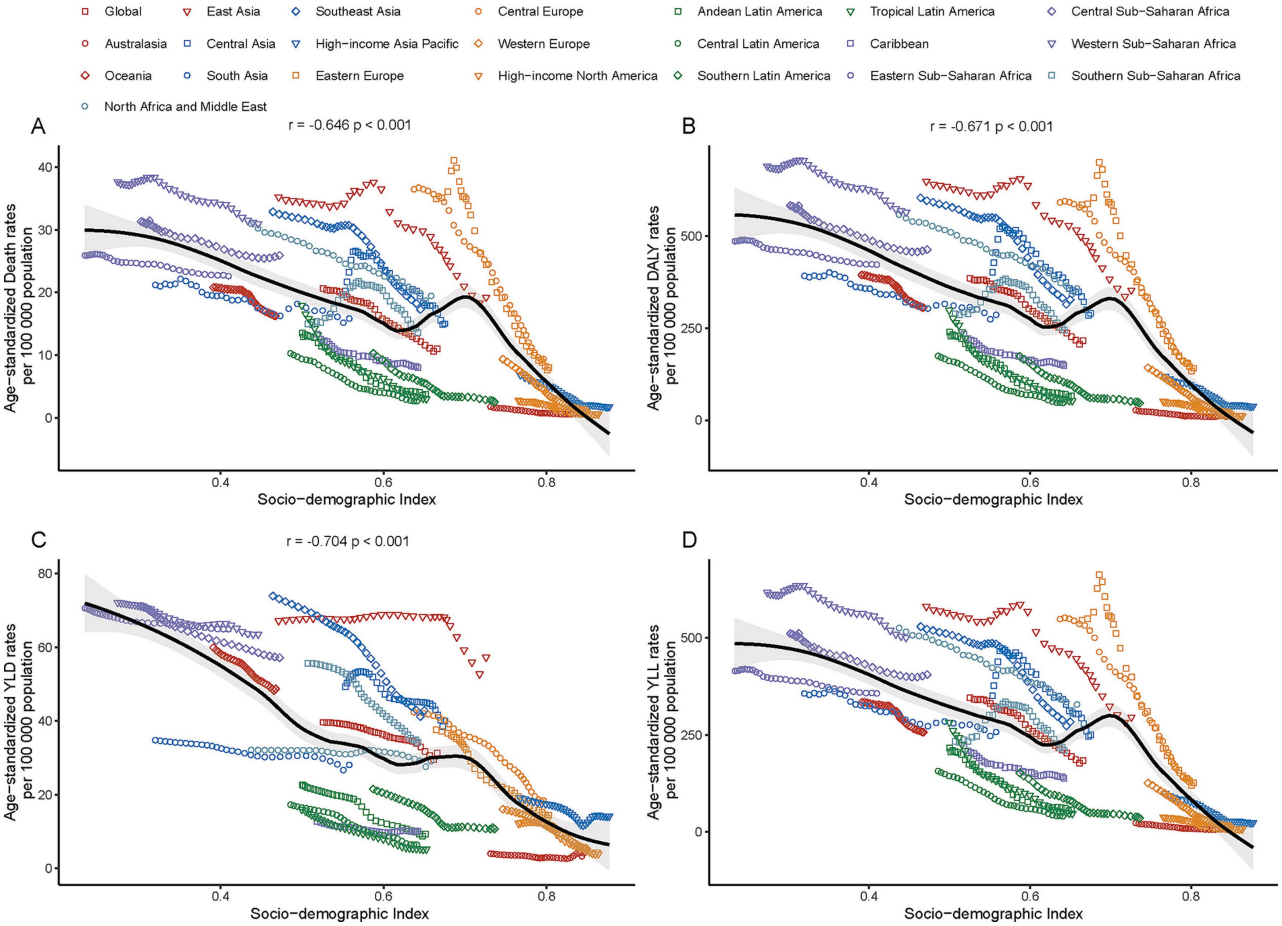
Correlation between age-standardized mortality rates **(A)** DALYs **(B)** YLLs **(C)** and YLDs **(D)** for ischemic stroke linked to particulate matter pollution and the Socio-demographic Index across Global Burden of Disease (GBD) regions in 2021.

In 2021, a significant negative correlation was observed between the SDI level and the age-standardized rates of deaths, DALYs, YLDs, and YLLs across 21 GBD regions (*r* = −0.74, *p* < 0.001; *r* = −0.76, *p* < 0.001; *r* = −0.76, *p* < 0.001; *r* = −0.66, *p* < 0.001) (Figure 3). The age-standardized rates of ischemic stroke burden attributable to total PMP generally decreased as the SDI increased. However, between SDI values of approximately 0.62–0.7, there was a brief increase in these rates before they declined again (Figure 4).

### 3.4 Global disease burden of ischemic stroke attributable to PMP from 1990 to 2021 by countries

**Figure 5:**
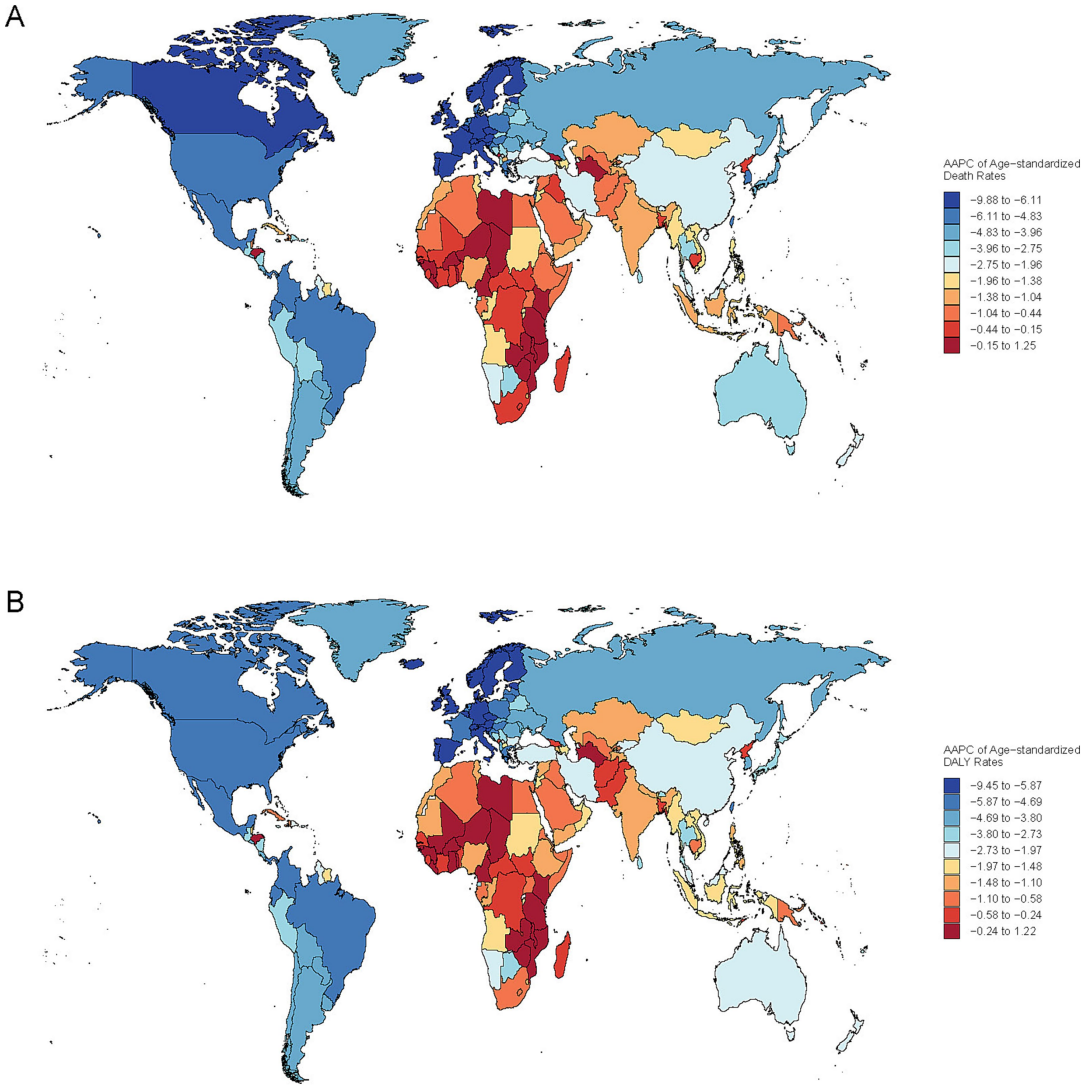
Global distribution of the average annual percent change (AAPC) in Age-Standardized Death **(A)** and DALYs **(B)**. Rates from ischemic stroke associated with total particulate matter pollution, 1990–2021.

**Figure 6:**
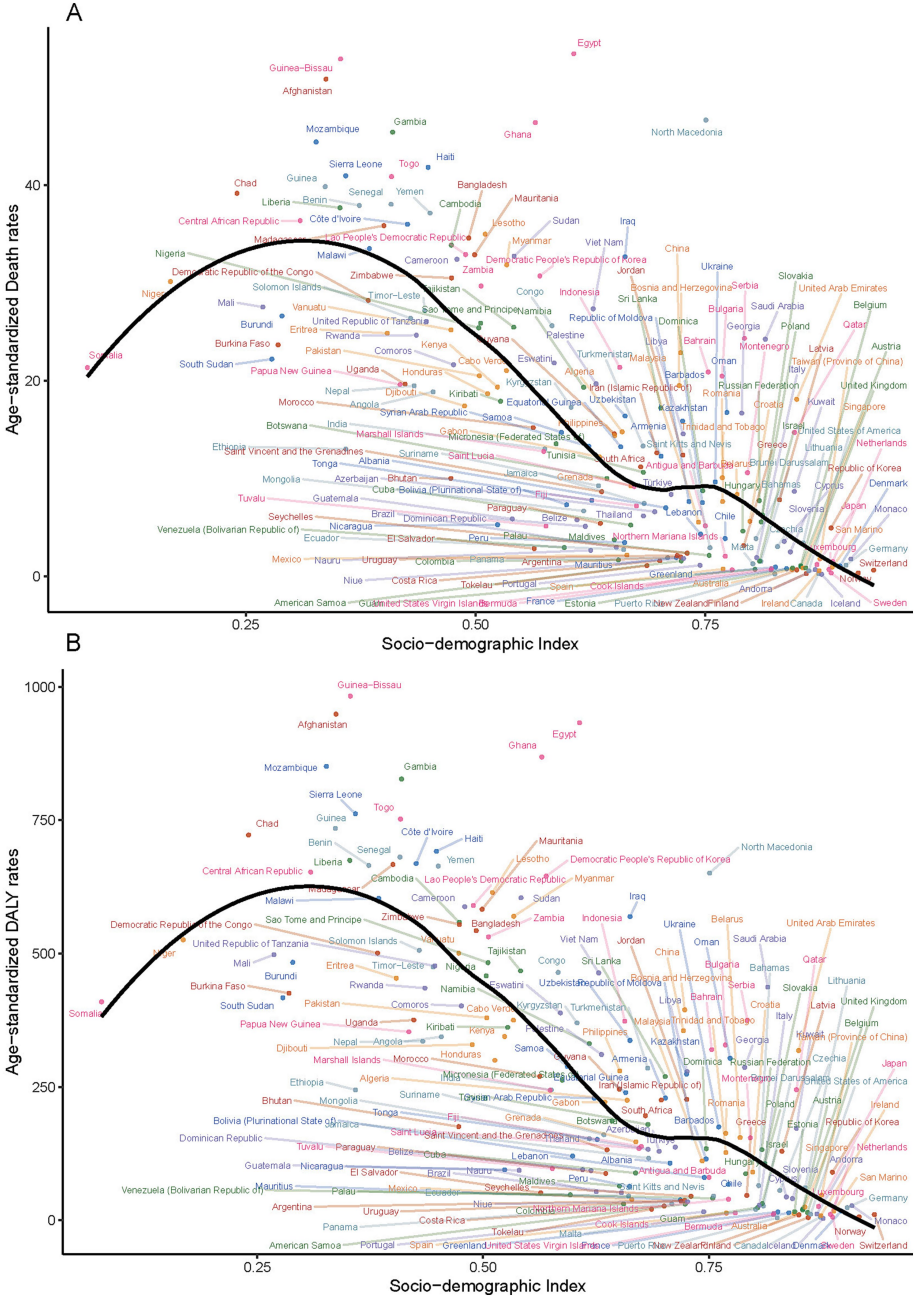
Correlation between age-standardized mortality rates and DALYs for ischemic stroke linked to particulate matter pollution and the Socio-demographic Index across 204 countries in 2021 **(A)** Age-standardized mortality rates, **(B)** Age-standardized DALYs rate.

In 2021, China reported the highest numbers of deaths, DALYs, YLDs, and YLLs attributable to ischemic stroke due to PMP, with approximately 357,000 deaths (95% UI: 257,600–472,460), 7.1 million DALYs (95% UI: 5,143,360–9,309,020), 1.2 million YLDs (95% UI: 796,990–1,684,360), and 5.9 million YLLs PMP (95% UI: 4,224,270–7,884,670). At the same year, there were 96 countries with ASDRs for ischemic stroke due to total PMP above the global level of 11.01 (95% UI: 8.44–13.96). The majority of these countries are located in Africa and Asia (Supplementary Table S4). The highest ASDRs for ischemic stroke related to total PMP were observed in several African countries (e.g., Egypt, Guinea-Bissau, Ghana, Gambia, Mozambique, Sierra Leone, Togo), as well as in Europe (North Macedonia) and the Caribbean region of North America (Haiti) (Supplementary Table S4). Conversely, the lowest ASDRs for ischemic stroke related to total PMP were observed in Europe (e.g., Iceland, Finland, Sweden, Norway, Ireland, Switzerland) and North America (e.g., Puerto Rico, Canada, United States of America, Bermuda) (Supplementary Table S4). Similar patterns appeared in age-standardized DALYs, YLDs, and YLLs (Supplementary Tables S4–S7). From 1990 to 2021, the AAPC in most countries showed a decreasing trend in the burden of ischemic stroke due to total PMP (Figure 5 and Supplementary Figure S7). Estonia exhibited the largest decreasing trend, with an AAPC for both death rates at −9.88 (95% CI: −10.63 to −9.13) and DALYs at −9.45 (95% CI: −10.12 to −8.77). The Maldives showed the largest decreasing trend for YLD with an AAPC of −6.2 (95% CI: −6.35 to −6.05), while Singapore recorded the largest decreasing trend for YLL with an AAPC of −9.1 (95% CI: −10.41 to −7.77) (Supplementary Tables S4–S7). A negative correlation was observed between the SDI level and the age-standardized rates of deaths, DALYs, YLDs, and YLLs across 204 countries (*r* = −0.74, *p* < 0.001; *r* = −0.76, *p* < 0.001; *r* = −0.76, *p* < 0.001; *r* = −0.75, *p* < 0.001) (Figure 6 and Supplementary Figure S8).

### 3.5 Global disease burden of ischemic stroke attributable to PMP from 1990 to 2021 by age and sex

**Figure 7:**
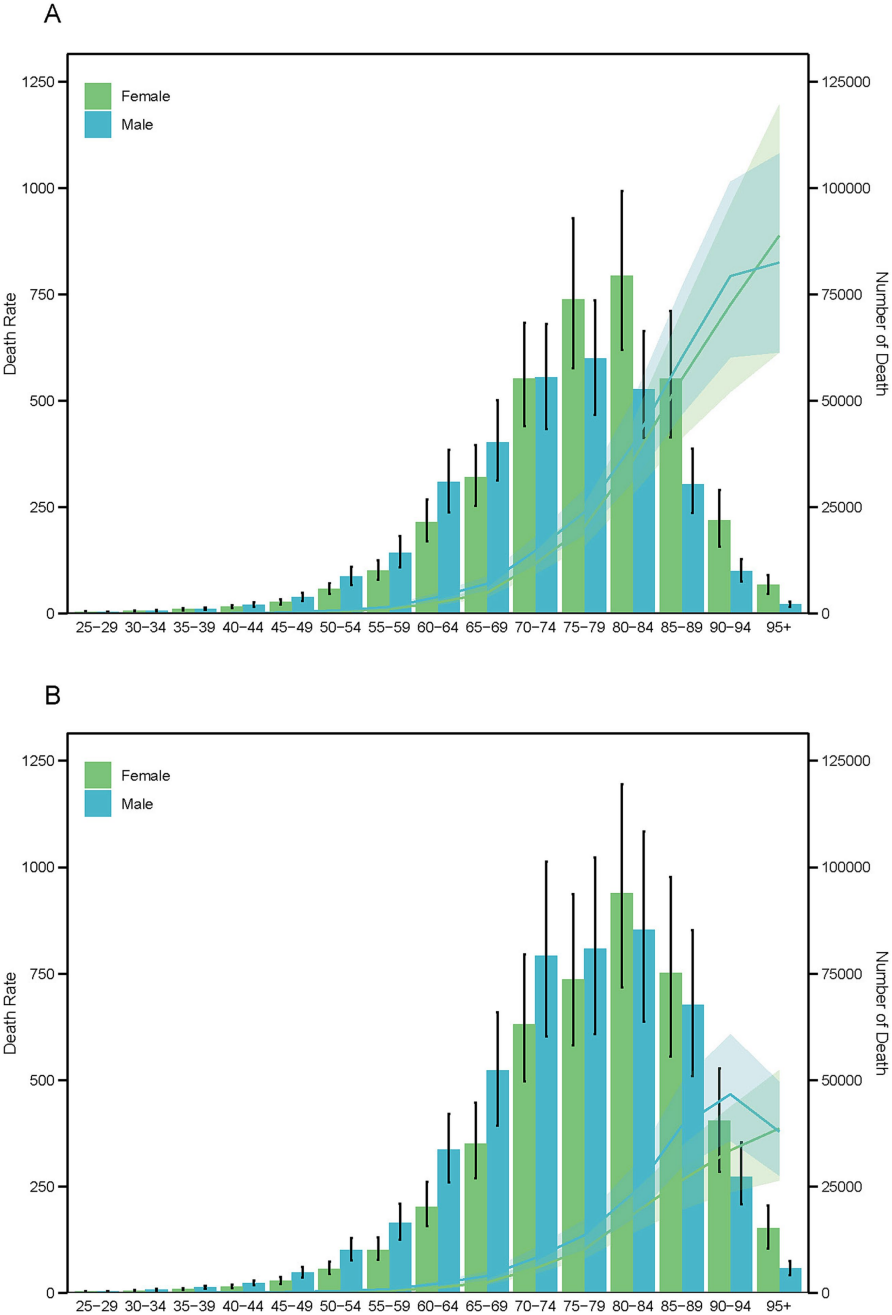
Age-specific death number (bar chart) and age-adjusted death rates (line chart) due to particulate matter pollution for the years 1990 **(A)** and 2019 **(B)**, categorized by sex.

In 1990, ischemic stroke mortality attributable to PMP was higher in males than in females up to the age group of 70–74 years. Peak mortality occurred at 75–79 years for males (237.78, 95% UI: 185.23–291.53) and at 80–84 years for females (359.06, 95% UI: 280.34–449.40). By 2021, this pattern had shifted: male mortality rates remained higher up to the age group of 75–79 years, and both genders peaked at 80–84 years (males: 232.85, 95% UI: 173.89–295.61; females: 184.30, 95% UI: 140.85–234.65) (Figure 7).

DALYs followed a slightly different trend. In 1990, males had higher DALYs than females up to the age group of 65–69 years, and this extended to 75–79 years by 2021. Post-peak ages saw a reversal: females peaked at 75–79 years in 1990 (3,500.10, 95% UI: 2,763.58–4,370.90) and at 70–74 years in 2021 (1,328.08, 95% UI: 1,048.42–1,673.67), while males peaked at 70–74 years in both years.

Female death and DALY rates increased linearly in both 1990 and 2021, whereas male death rates decelerated at ages 90–94 years, and DALY rates decelerated at ages 85–90 years in 2021 and 90–94 years in 1990 (Supplementary Figure S9). YLLs showed a similar pattern to deaths, while YLDs exhibited a distinct trend. Females consistently had higher YLDs than males across all age groups, peaking at 65–69 years in both 1990 and 2021 (Supplementary Figures S10, S11). Males, however, shifted their peak YLDs from 65 to 69 years in 1990 to 70–74 years in 2021. The YLD rate between females and males followed a gradual reverse U-shaped trend (Supplementary Figure S10).

## 4 Discussion

We studied the trends of PMP globally and in various nations. From 1990 to 2021, ischemic stroke deaths and disease burden linked to total PMP increased, but the age-standardized mortality rate decreased. Global projections for 2022 to 2050 suggest that age-standardized rates of deaths, DALYs, YLLs, and YLDs connected to ischemic strokes and total PMP will keep rising. The role of APMP in ischemic strokes is becoming more significant, while HAP’s impact is reducing. High-middle and middle SDI regions faced heavier health burdens, but age-standardized indicators generally declined across all SDI regions. Low SDI was mainly affected by HAP, while high-middle and high SDI were more impacted by APMP. East Asia, South Asia, and Southeast Asia had the highest ischemic stroke burden due to PMP in 2021. High SDI regions, like Western Europe and North America, mainly attributed their ischemic strokes to APMP. China had the highest ischemic stroke burden due to PMP in 2021. Also, 96 countries had higher age-standardized ischemic stroke mortality rates than the global average, mostly in Africa and Asia. Male mortality rates due to ischemic strokes from PMP were higher than females only in older age groups, while females had higher YLDs across all ages. A negative correlation was found between SDI levels and the stroke burden across 204 countries and 21 GBD region.

### 4.1 Epidemiological study of ischemic stroke related to PMP

Over the past three decades, epidemiological studies, including meta-analyses, have unequivocally demonstrated that the primary cause of disease and death linked to air pollution exposure is heart disease, rather than lung ailments. Notably, several investigations have substantiated a close association between long-term exposure to air pollution and the incidence of strokes. For instance, the Rome longitudinal study revealed an 8% increase in stroke mortality for every 10 μg/m^3^ elevation in PM_2.5_ (21). The ESCAPE study indicated a 19% rise in stroke mortality per 5 μg/m^3^ increment of PM_2.5_ (22). A meta-analysis conducted in 2013 showed that for every 10 μg/m^3^ increase in PM_2.5_, there was a 6% increase in the risk of all-cause mortality and an 11% increase in the risk of cardiovascular mortality (23). Similarly, a 2014 study found that for every 10 μg/m^3^ increase in NO_2_ concentration, there was a 13% increase in cardiovascular mortality (24).

Furthermore, a comprehensive meta-analysis encompassing 28 countries and over 6.2 million events underscored a significant association between stroke admissions or deaths and increased PM_2.5_ concentrations. Specifically, for every 10 μg/m^3^ rise in PM_2.5_, the hazard ratio for stroke admission or death escalated by 1.011 (25). The study also highlighted a stronger correlation in low- and middle-income countries, suggesting the need for policy reforms in these highly polluted regions to reduce individual exposure to air pollutants. Globally, 716 million people living on less than $1.90 per day are directly exposed to unsafe PM_2.5_ concentrations. Among them, 405 million (57%) reside in Sub-Saharan Africa, and 275 million are exposed to harmful PM_2.5_ levels. Countries where poverty and unsafe air pollution coexist also score low in terms of healthcare accessibility and quality, thereby exacerbating vulnerability. Approximately one in 10 people exposed to unsafe air pollution levels lives in extreme poverty (26). Dominici et al. (67) discovered that for every 10 μg/m^3^ increase in same-day PM_2.5_, there was a 0.81% rise in hospital admissions due to cerebrovascular disease in the United States (27). Leiva et al. (28) identified that in Chile, where the mean PM_2.5_ concentration is over twice as high at 31 μg/m^3^, cerebrovascular disease hospitalizations increased by 1.29% for every 10 μg/m^3^ increment. Additionally, Lokken et al. (7) observed that in cases of acute ischemic stroke, symptoms often manifest the day before hospital admission. This delay in seeking medical attention could potentially underestimate the overall association between pollution exposure and strokes.

### 4.2 Physiological mechanisms linking air pollution and stroke

Although the specific physiological mechanisms linking air pollution and stroke have not been fully elucidated, research indicates that air pollution can trigger several physiological responses associated with stroke risk (29). The first possible mechanism involves inflammation, oxidative stress, and lipid modification. Inhalation of particulate matter induces lung inflammation, and inflammatory factors enter the systemic circulation, indirectly affecting the cardiovascular system. This inflammation, combined with oxidative stress, amplifies the pathophysiological effects of pollutants. Oxidative stress reduces the availability of nitric oxide (NO) in vascular endothelium, impairing endothelium-dependent vasodilation, and can lead to elevated blood pressure. Additionally, particles promote low-density lipoprotein oxidation and the production of atherosclerotic factors, further damaging endothelial function (30–32). The second mechanism involves the translocation of nanoparticles. Due to their small size, these particles can cross the alveolar-capillary barrier into the systemic circulation, directly affecting blood vessels and circulating blood cells (33). They activate pro-inflammatory responses in endothelial cells and upregulate adhesion molecules, promoting atherosclerosis (34, 35). Nanoparticles also disrupt tight junctions between endothelial cells, increasing permeability and leading to endothelial barrier dysfunction (36). Moreover, these particles might enter the central nervous system via the nasal route, affecting neurological or vascular systems (37, 38). The third mechanism is vasoconstriction and autonomic dysfunction. Inhalation of particles or subsequent lung inflammation can stimulate nerve receptors on alveolar surfaces, altering autonomic functions and affecting cardiovascular stability (39). Numerous studies show that PM_2.5_ exposure reduces heart rate variability, a marker of cardiac autonomic dysfunction, associated with poor outcomes in heart disease patients and the general population (40, 41). Exposure to urban and diesel exhaust particles increases arrhythmia incidence, linking air pollution to cardioembolic stroke (42, 43). The fourth mechanism, thrombogenicity, involves findings that inhalation of diluted diesel exhaust enhances platelet activation and coagulation. This potentially explains the association between combustion-related air pollution and acute cardiovascular events. Further research is needed to clarify the specific mechanisms leading to increased thrombosis (44). The fifth mechanism is atherosclerotic plaque instability. Pollutants promote the formation, progression, and rupture of atherosclerotic plaques via oxidative stress and inflammatory pathways, leading to vascular injury and endothelial activation and stimulating monocyte migration to vascular sites (29). Long-term pollutant exposure relates to increased plaque instability, higher lipid content, vascular inflammation, and oxidative stress, potentially worsening intracranial atherosclerosis. Acutely, these cellular responses can trigger ischemic stroke onset, while chronically, they may contribute to cerebrovascular disease risk factors such as diabetes, hypertension, cardiac arrhythmia, and accelerated atherosclerosis.

### 4.3 The role of population aging and environmental factors in increasing stroke burden

Even though between 1990 and 2021, deaths and DALYs from ischemic stroke attributable to total PM exposure increased, the corresponding age-standardized rates and AAPC showed decreases. However, global projections from 2022 to 2050 reveal a continuous rise in age-standardized rates of deaths, DALYs, YLLs, and YLDs attributable to ischemic stroke linked to total PMP. Notably, the global figures for deaths, DALYs, YLLs, and YLDs associated with both APMP and HAP have surpassed those of the past 5 years. This upward trend could be partly explained by global population growth and aging, which have contributed to a higher incidence of stroke. As the population ages, there is an escalation in the prevalence of risk factors such as hypertension, diabetes, and obesity, which are further exacerbated by air pollution. The United Nation’s medium variant predicts a 47% increase in the population of less developed regions by 2,100, from 6.5 billion to 9.6 billion, compared to a 2% decrease in more developed regions (45). This population growth exacerbates risks like flooding and water stress, particularly in less developed countries where people are more vulnerable to climate risks and disproportionately exposed to climate impacts (46). As the population ages, there is an escalation in the prevalence of risk factors such as hypertension, diabetes, and obesity, which are further exacerbated by air pollution. Statistical analysis shows that older populations have higher risk estimates for particulate matter-related total and stroke mortality compared to younger populations (47). According to a United Nations report, the population aged 65 and older is expected to double from 0.7 billion in 2019 to 1.5 billion in 2050 (48). This demographic shift necessitates significant healthcare reforms to address a projected 55% increase in global disability-adjusted life years among those aged 60 and older by 2030 (49). Furthermore, between 1990 and 2017, the global proportion of individuals aged 65 and older rose from 6.1 to 8.8%, accounting for 27.9% of total global deaths in 2017. The primary disease-specific contributions to deaths from population aging during this period were ischemic heart disease and stroke, resulting in 3.2 million and 2.2 million deaths, respectively (50). Alongside air pollution, high temperatures have also been linked to an increased risk of stroke. The contribution of high temperatures to stroke-related health issues has risen significantly, indicating that environmental factors play a crucial role in the growing burden of stroke (51).

### 4.4 Differential impact of APMP and HAP on the global stroke burden analysis

Moreover, our study found that between 1990 and 2021, the global proportion of the ischemic stroke burden attributable to APMP increased to 66.6%, while the contribution from HAP decreased to 33.4%.

This trend was particularly evident in high SDI regions, where APMP was the primary factor. This trend may be attributed to the continuous decline in household PM_2.5_ levels over recent decades in high SDI regions, driven by advancements in combustion technology and a global shift toward cleaner energy sources like liquefied petroleum gas and electricity (52). Consequently, APMP from persistent sources inherent to high-SDI economies—such as transportation, industry, energy production, and intensive agriculture—becomes the predominant and more intractable air pollution challenge (53). The burden of stroke related to air pollution was disproportionately higher in low- and middle-income countries, and predominantly linked to HAP. According to the study, in 2010, Africa and Southeast Asia had the highest proportions of households using solid fuels at 77 and 61%, respectively, while Europe and the Americas had the lowest usage at under 20%. Notably, the Western Pacific and Eastern Mediterranean regions were mid-range, and high-income countries reported less than 5% usage, with Asia experiencing the most significant declines in usage. Furthermore, by 2010, while Africa and the Eastern Mediterranean saw increases in solid fuel use exposure, with Africa reaching a 77% prevalence, Southeast Asia experienced a decline in prevalence from 95 to 61% despite stable exposure numbers due to population growth. This contrasts with Europe, the Americas, and the Western Pacific, where both prevalence and exposure declined (54).

### 4.5 Global disparities in stroke burden and environmental performance: challenges and progress

In 2021, 96 countries, mostly in Africa and Asia, had a ASDRs from PMP related to ischemic stroke higher than the global average. The 2024 Environmental Performance Index reveals that Vietnam and several other developing nations in Southeast and Southern Asia, including Pakistan, Laos, Myanmar, and Bangladesh, rank lowest in terms of greenhouse gas emissions. This underscores the pressing need for international collaboration to pave a path toward sustainability for these struggling countries (55). Conversely, European countries such as Iceland, Finland, Sweden, Norway, Ireland, and Switzerland exhibited the lowest ASDRs for ischemic stroke linked to overall PMP. Notably, Estonia, Finland, Greece, Timor-Leste, and the United Kingdom have successfully reduced their GHG emissions over the past 10 years, aligning with the rate required to attain net-zero emissions by 2050. Estonia demonstrated the most significant downward trend in both death and DALYs, with an AAPC. Since 1990, Estonia has impressively cut its greenhouse gas emissions by 59%, according to the 2024 Environmental Performance Index. Looking ahead, the energy sector is poised to play a pivotal role in further emission reductions, aiming to achieve 100% renewable electricity consumption by 2030. Over the past decade, emissions have dropped by 40%, primarily due to the shift from oil shale power plants to cleaner energy sources. Currently, Estonia is developing plans to establish a CO2-neutral energy sector and public transport network in major cities by 2040 (55). In 2021, China reported the highest numbers of deaths, DALYs, YLDs, and YLLs attributable to ischemic stroke due to PMP, with approximately 357,000 deaths, 7,095,510 DALYs, 1,187,750 YLDs, and 5,907,760 YLLs. In recent decades, China has made significant strides in mitigating atmospheric pollutant emissions, as evidenced by data published by the Ministry of Ecology and Environment. This progress is reflected in the improved air quality observed in most of the country’s cities, where standard pollutants now adhere to established standards and guidelines. Despite these advancements, PM_2.5_ continues to pose a significant challenge, particularly in regions characterized by dense industrial activity and high population density (56).

### 4.6 Age and sex differences in the impact of air pollution on ischemic stroke risk

From 1990 to 2021, the impact of PMP on ischemic stroke mortality reveals notable age and sex-related differences. Specifically, ischemic stroke mortality rates due to PMP are higher in males only at more advanced ages, and peak mortality rates have increased for both sexes during this period. Additionally, females consistently exhibit higher YLDs across all age groups than males, while trends in DALYs show different growth patterns for both sexes. Males exhibited consistently higher mortality rates, while females bore a greater burden of YLDs. This aligns with broader global health trends where males experience higher premature mortality and lower life expectancy, whereas females often survive longer but with higher morbidity and poorer health-related quality of life in later years. The research suggests that as people age, the mortality and incidence rates associated with pollution exposure increase. This is primarily because the older adult have a heightened susceptibility to the health impacts of air pollution due to the decline in physiological functions and the high prevalence of chronic diseases (57). In 2019, globally, the age – standardized mortality rate of males due to air pollution was 1.5 times higher than that of females, and this gender disparity was present across most age groups (58). Chen et al. (68) and Shin et al. (60) discovered that exposure to specific PM_2.5_ components was associated with a higher mortality rate in males, potentially attributable to their smoking habits (59, 60). However, studies in Japan and other regions found that there was a significant correlation between PM_2.5_ exposure and asthma in females, while no such association was observed in males (61). These differences highlight the complex interplay between sex, age, and stroke type incidence. A study from Canada indicated that women under 30 have a higher risk of ischemic stroke compared to men, whereas middle-aged men are at higher risk than women (62). By the age of 80, stroke risk becomes more equal between genders, but in those above 85, women might experience a higher incidence than men (63, 64). A scoping review in Cureus highlights that older individuals and those with pre-existing conditions such as diabetes are particularly vulnerable to the negative effects of particulate matter exposure. Interestingly, studies examining the sex influence on PM-related stroke risk find inconsistent results; some suggest a stronger association in women, while others indicate no significant differences (65). However, a specific longitudinal study involving 155,410 postmenopausal women over 15 years demonstrated that long-term exposure to fine particulate matter (PM_2.5_, PM_10_) and nitrogen dioxide (NO_2_) significantly increases the risk of cerebrovascular events. Notably, the strength of these associations was consistent across different stroke etiologies (66). Those insights underscore the need for nuanced evaluation of environmental risk factors in stroke prevention strategies, considering gender and age-specific differences. Public health policies should focus on improving air quality to mitigate stroke risks, particularly in vulnerable populations such as older adults and those with pre-existing health conditions.

### 4.7 Limitations

(1) Data quality and heterogeneity: the reliance on the GBD 2021 database introduces potential biases due to variations in data completeness and accuracy across regions, particularly in low- and middle-income countries where surveillance systems may be underdeveloped. For instance, misclassification of ischemic stroke subtypes (e.g., underreporting of HAP-related cases in regions with limited household air pollution monitoring) could skew burden estimates. Additionally, PM_2.5_ exposure assessments, derived from modeled estimates rather than direct individual-level measurements, may not fully capture localized or microenvironmental variations in pollution exposure. The pollution does not contain PM_2.5_; it also includes other pollutants like PM₁₀, NO₂, O₃, and SO₂. These pollutants can either act independently or in combination to affect the disease burden. Although the GBD 2021 has achieved some progress in incorporating pollutant data, the combined and interactive impacts of multiple pollutants on disease burden assessment remain largely unexplored. (2) Model Assumptions and Residual Confounding: The BAPC model assumes linear additive effects of age, period, and cohort, which may oversimplify complex interactions between demographic shifts and pollution exposure dynamics. While the model accounts for socio-demographic factors via SDI, residual confounding from unmeasured variables (e.g., genetic predisposition, access to acute stroke care, or co-exposure to other pollutants) could influence the observed associations. Furthermore, Joinpoint regression assumes abrupt trend changes at specific years, potentially masking gradual or non-linear transitions in pollution policies or healthcare interventions. (3) Projection uncertainties: long-term projections (2022–2050) are contingent on the stability of current trends in pollution control, population aging, and healthcare infrastructure—factors that may be disrupted by unforeseen events (e.g., pandemics, geopolitical conflicts, or climate crises). The BAPC model’s reliance on WHO population weights also assumes uniform age-structure standardization, which may not reflect future demographic disparities. Finally, while the study highlights shift in APMP- and HAP-related burdens, it does not explicitly quantify the contribution of overlapping or synergistic effects between these two PMP subtypes, limiting policy-specific insights.

## 5 Conclusion

This global analysis reveals a critical dual burden of ischemic stroke attributable to PMP: APMP has emerged as the dominant driver in high- and middle-income regions, HAP remains a persistent threat in low-resource settings. Despite a 46.65% decline in global age-standardized death rates (1990–2021), rising absolute numbers of deaths and DALYs underscore the urgency of pollution control amidst population growth and aging. Striking disparities were observed: APMP-related burdens peaked in high-SDI regions (e.g., Western Europe) but are now shifting to middle-SDI countries (e.g., China and South Asia), while HAP disproportionately affects sub-Saharan Africa.

The study advances methodological rigor by integrating BAPC modeling with Joinpoint regression, demonstrating that APMP’s impact is tightly coupled with industrial activity and urbanization, whereas HAP reflects delayed progress in clean energy transitions. Projections to 2050 warn of escalating burdens unless targeted interventions—such as APMP mitigation in rapidly urbanizing regions and accelerated HAP reduction in low-SDI nations—are prioritized.

Three imperatives emerge: (1) high/middle-SDI regions require stricter PM_2.5_ emission controls and climate-resilient urban planning, while low-SDI areas need subsidized clean cooking technologies. (2) Stroke prevention programs must incorporate real-time pollution exposure alerts, particularly for aging populations in high-burden regions. (3) Future studies should resolve PMP’s synergistic effects with non-communicable diseases and leverage individual-level exposure data to refine risk stratification.

By contextualizing PMP’s evolving role in cerebrovascular disease, this work provides a benchmark for evaluating the WHO’s Air Quality Guidelines and informs the UN Sustainable Development Goals (SDGs) on health equity and environmental sustainability.

## Data Availability

The datasets presented in this study can be found in online repositories. The names of the repository/repositories and accession number(s) can be found in the article/Supplementary material.

## Footnotes

^1^ http://ghdx.healthdata.org/gbd-results-tool

## Supplementary material

SUPPLEMENTARY FIGURE S1 Global DALYs from ischemic stroke due to particulate matter pollution (1990-2021), by Socio-demographic Index (SDI) levels, shown as numbers and age-standardized rates. Includes total, household, and ambient pollution.

SUPPLEMENTARY FIGURE S2 Global YLDs from ischemic stroke due to particulate matter pollution (1990-2021), by SDI levels, shown as numbers and age-standardized rates. Includes total, household, and ambient pollution.

SUPPLEMENTARY FIGURE S3 Global YLLs from ischemic stroke due to particulate matter pollution (1990-2021), by SDI levels, shown as numbers and age-standardized rates. Includes total, household, and ambient pollution.

SUPPLEMENTARY FIGURE S4 Contribution of DALYs (numbers and percentages) from household and ambient pollution, globally and by region (1990-2021).

SUPPLEMENTARY FIGURE S5 Contribution of YLDs (numbers and percentages) from household and ambient pollution, globally and by region (1990-2021).

SUPPLEMENTARY FIGURE S6 Contribution of YLLs (numbers and percentages) from household and ambient pollution, globally and by region (1990-2021).

SUPPLEMENTARY FIGURE S7 Global distribution of the Average Annual Percent Change (AAPC) in age-standardized YLD **(A)** and YLL **(B)** rates from ischemic stroke linked to total pollution (1990-2021).

SUPPLEMENTARY FIGURE S8 Correlation between age-standardized YLLs and YLDs for ischemic stroke due to pollution and the Socio-demographic Index across 204 countries in 2021.

SUPPLEMENTARY FIGURE S9 Age-specific DALYs (bar chart) and age-adjusted DALYs rates (line chart) due to pollution in 1990 **(A)** and 2019 **(B)**, by sex.

SUPPLEMENTARY FIGURE S10 Age-specific YLDs (bar chart) and age-adjusted YLDs rates (line chart) due to pollution in 1990 **(A)** and 2019 **(B)**, by sex.

SUPPLEMENTARY FIGURE S11 Age-specific YLLs (bar chart) and age-adjusted YLLs rates (line chart) due to pollution in 1990 **(A)** and 2019 **(B)**, by sex.
