# Supplementary figures and images for: Global patterns and trends in ischemic stroke burden attributable to particulate matter pollution: changes from 1990 to 2021 and projections from 2022 to 2050
Source: Front Public Health. 2025 Jun 26;13:1599541. doi: 10.3389/fpubh.2025.1599541 (PMC12241019; doi:10.3389/fpubh.2025.1599541)

High SDI Low SDI High-middle SDI Low-middle SDI Middle SDI

A

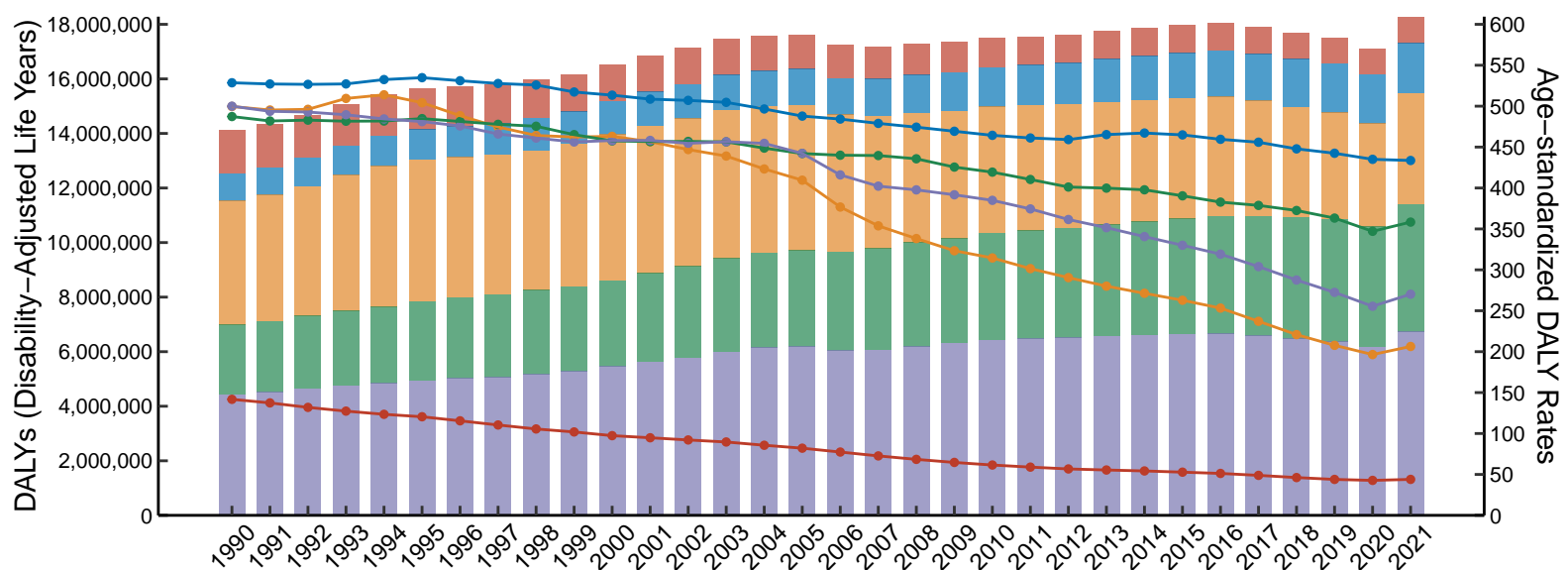

B

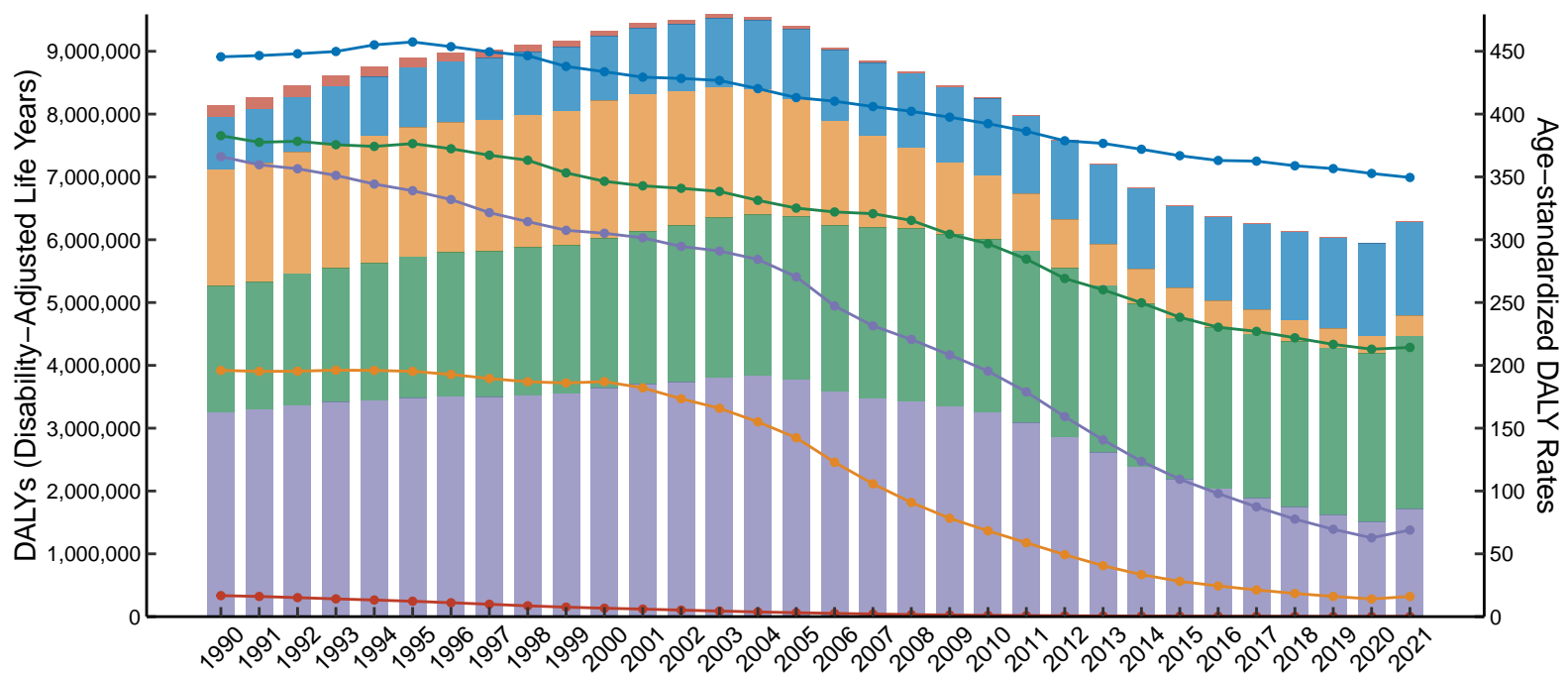

C

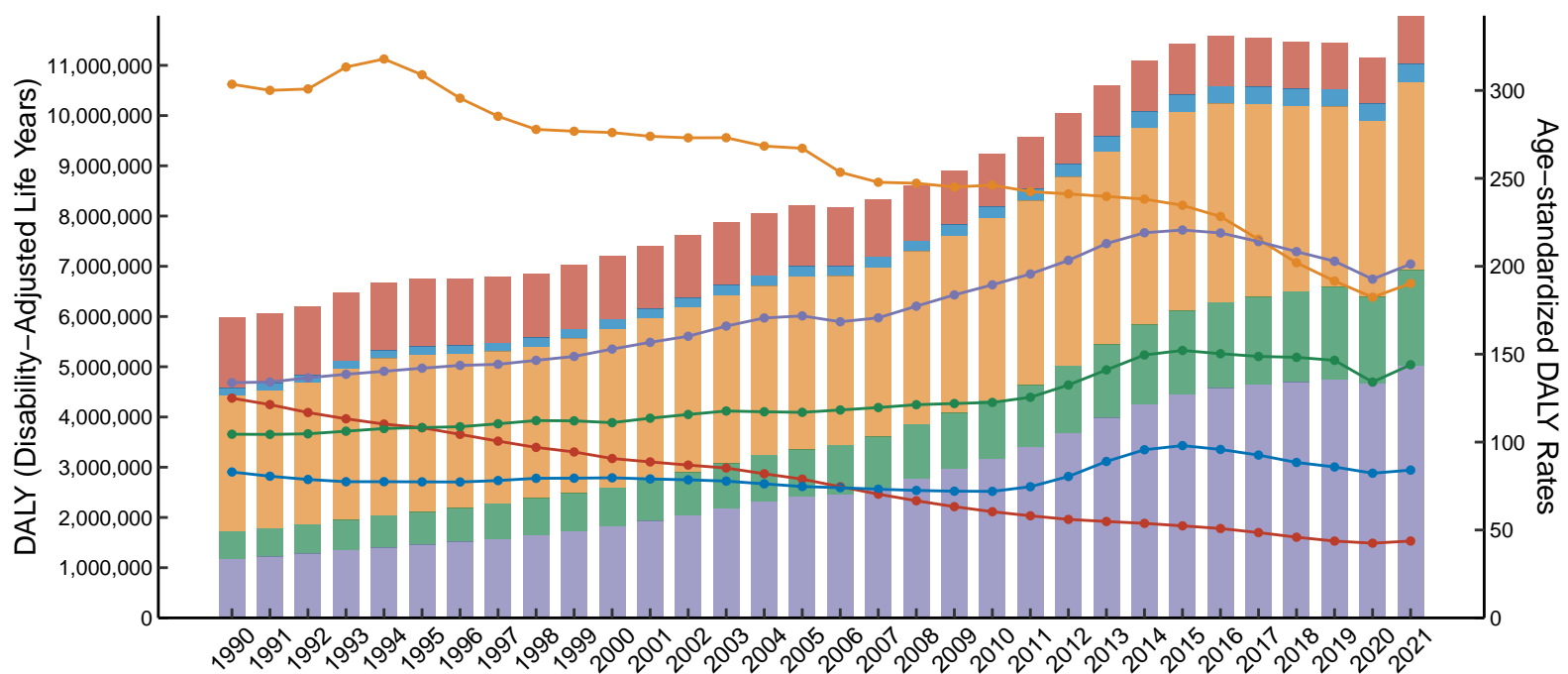

Supplement: SUPPLEMENTARY FIGURE S1 — Global DALYs from ischemic stroke due to particulate matter pollution (1990-2021), by Socio-demographic Index (SDI) levels, shown as numbers and age-standardized rates. Includes total, household, and ambient pollution. [file Data_Sheet_1.PDF]

High SDI Low SDI High-middle SDI Low-middle SDI Middle SDI

A

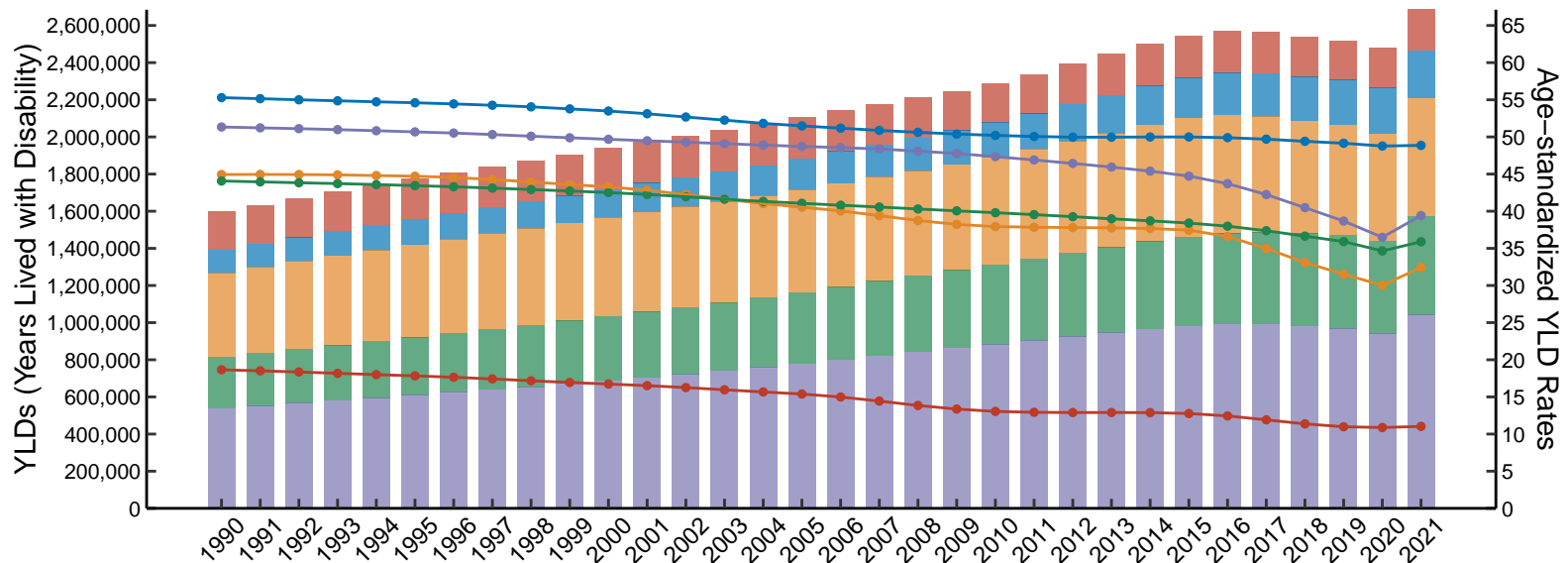

B

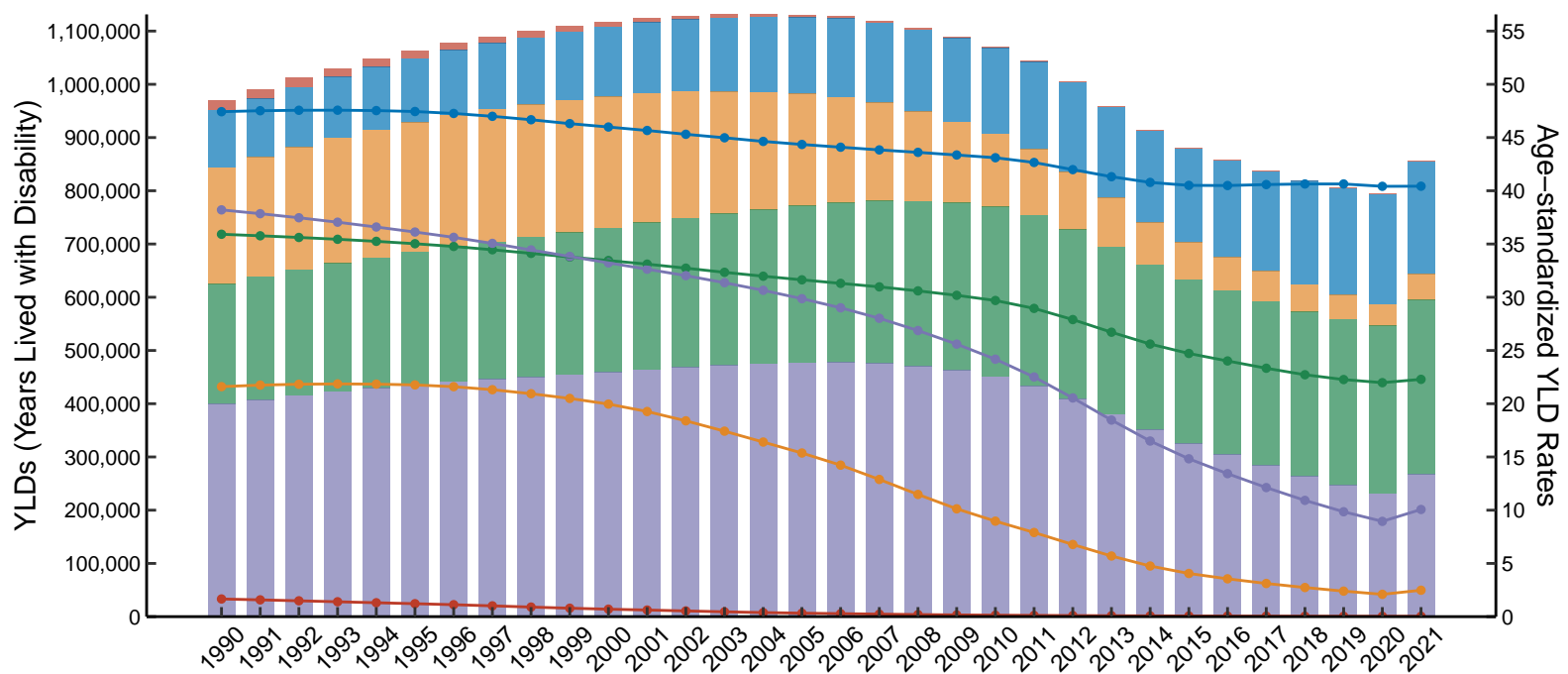

C

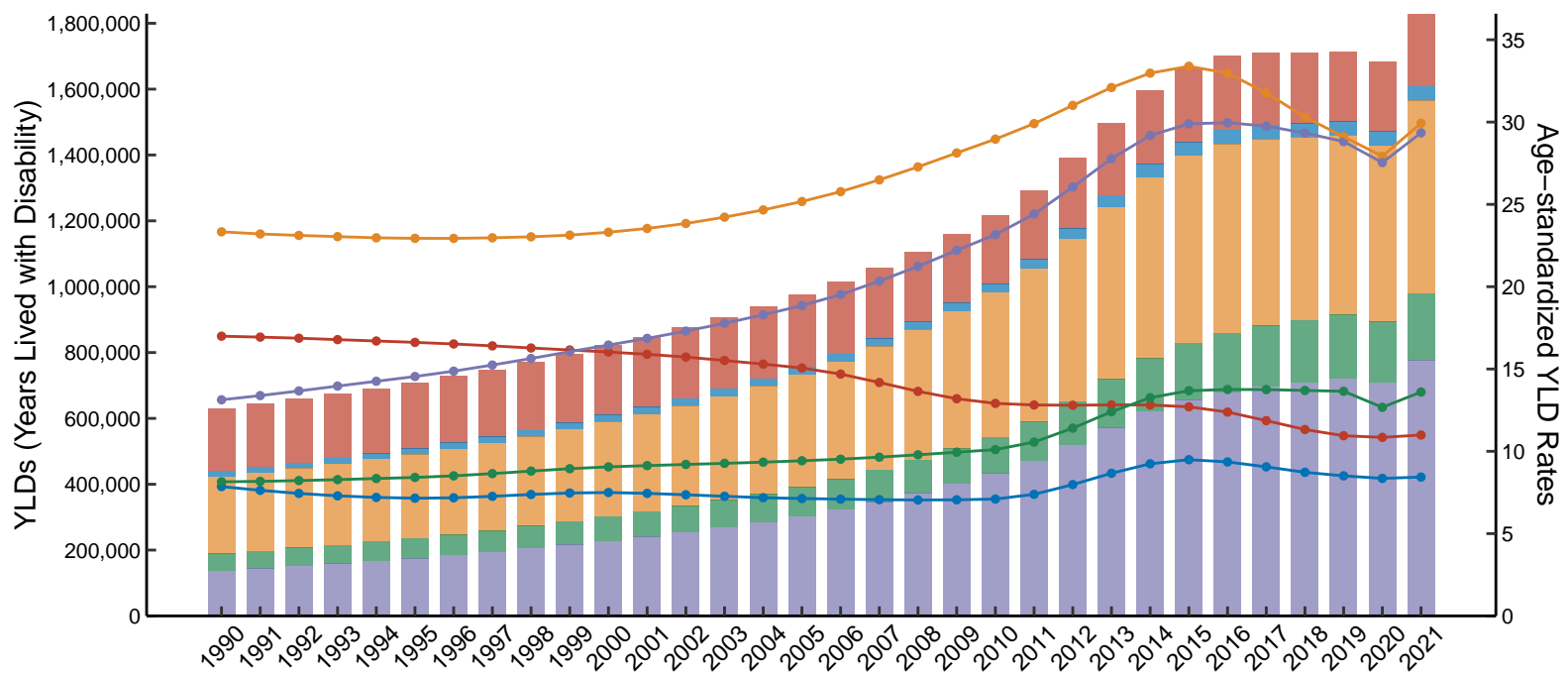

Supplement: SUPPLEMENTARY FIGURE S2 — Global YLDs from ischemic stroke due to particulate matter pollution (1990-2021), by SDI levels, shown as numbers and age-standardized rates. Includes total, household, and ambient pollution. [file Data_Sheet_2.PDF]

High SDI Low SDI High-middle SDI Low-middle SDI Middle SDI

A

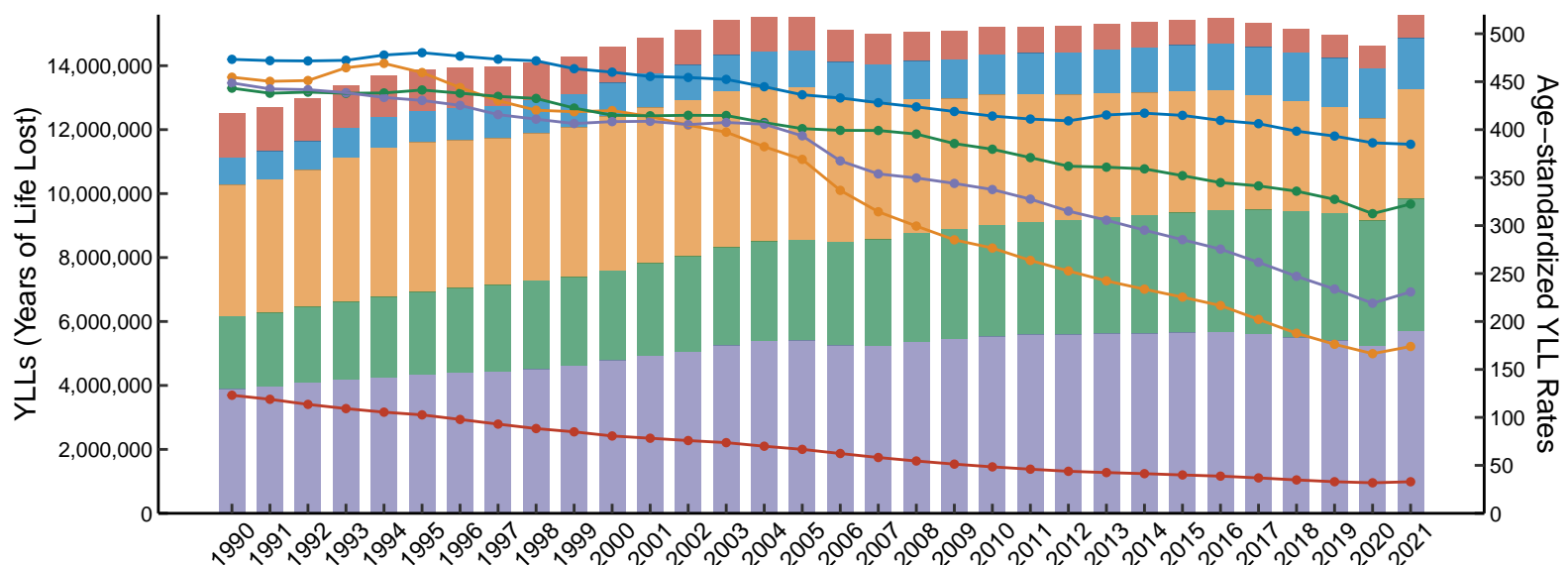

B

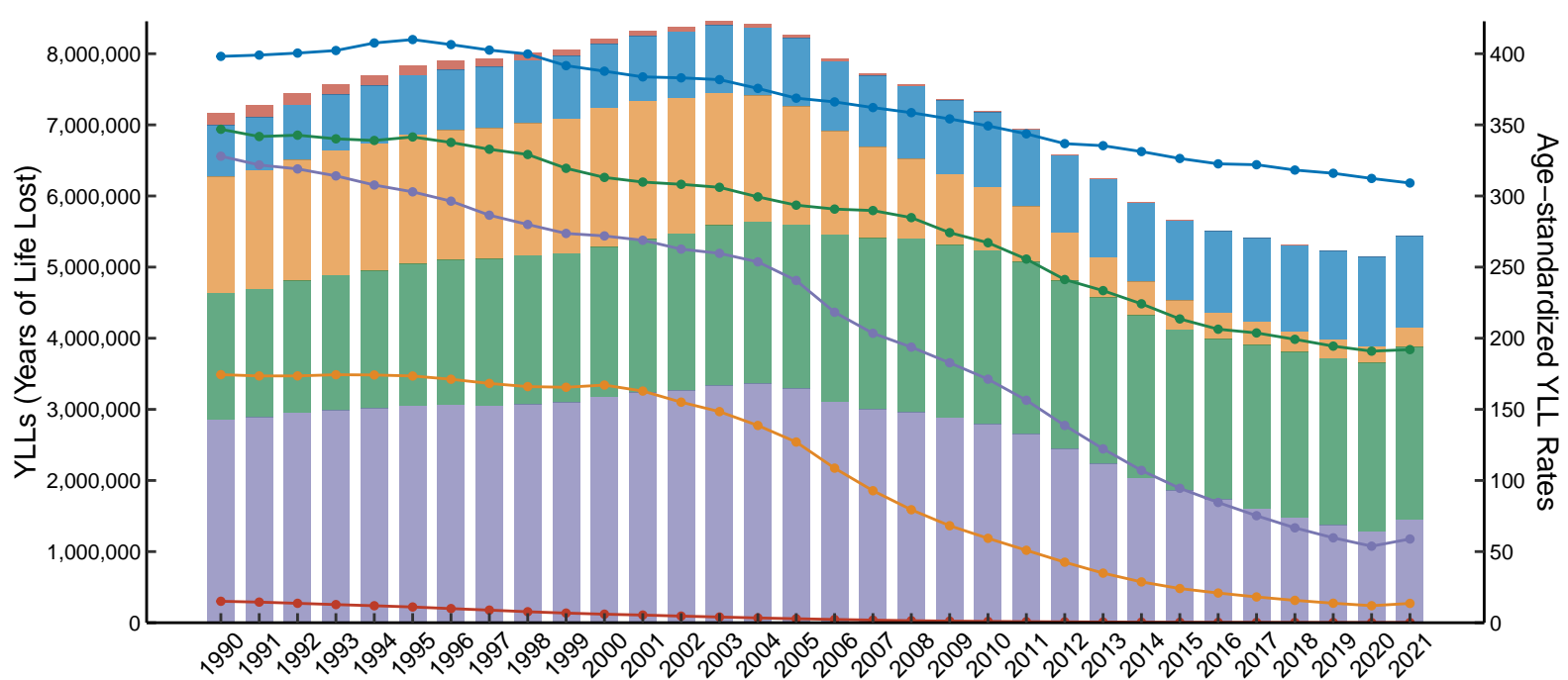

C

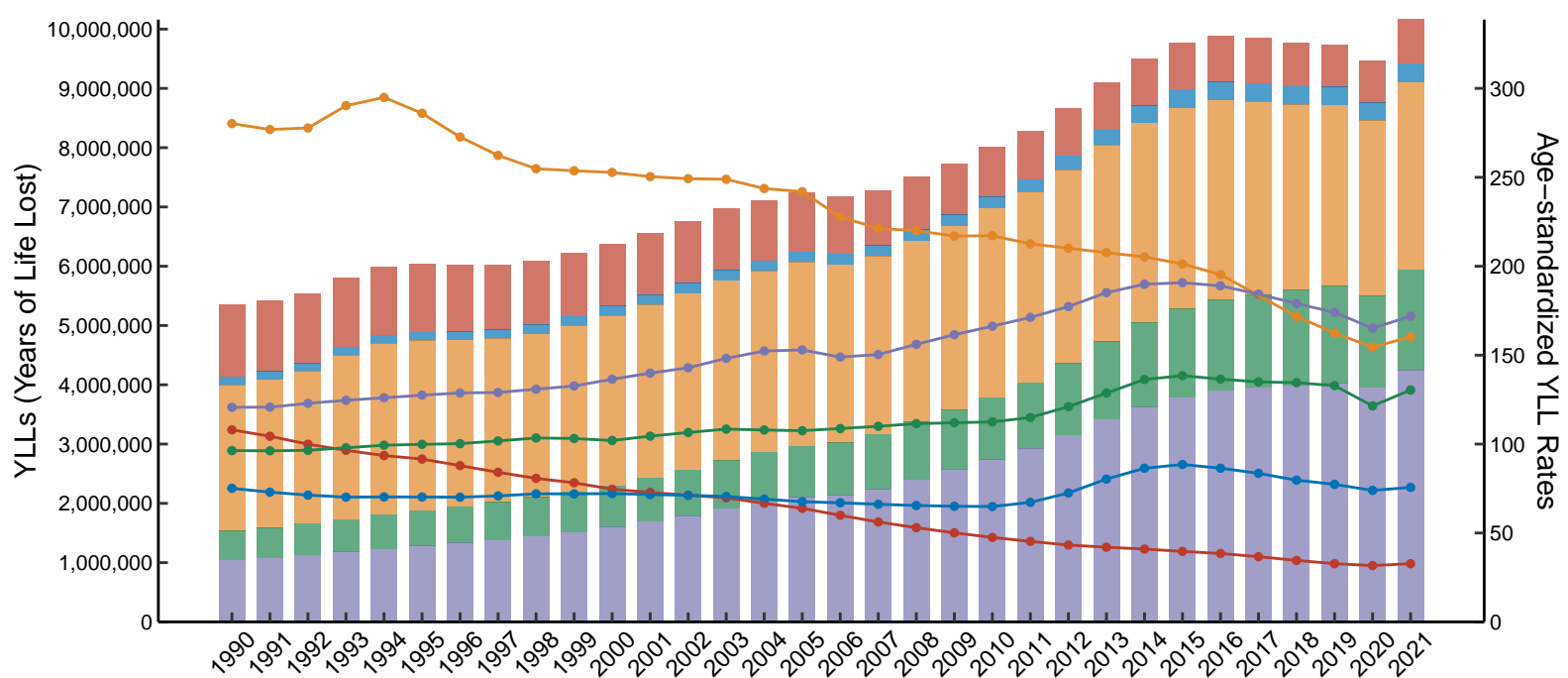

Supplement: SUPPLEMENTARY FIGURE S3 — Global YLLs from ischemic stroke due to particulate matter pollution (1990-2021), by SDI levels, shown as numbers and age-standardized rates. Includes total, household, and ambient pollution. [file Data_Sheet_3.PDF]

Ambient particulate matter pollution Household air pollution from solid fuels

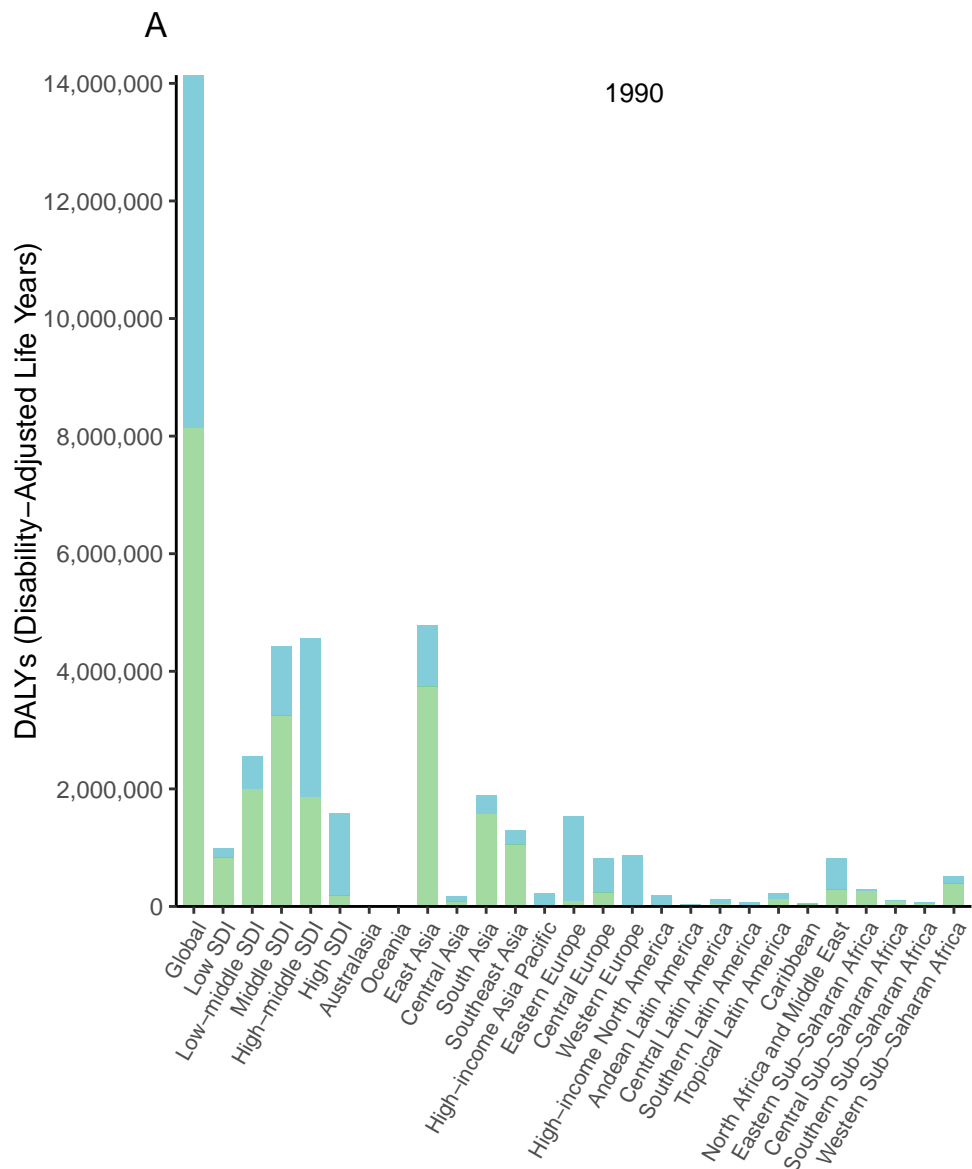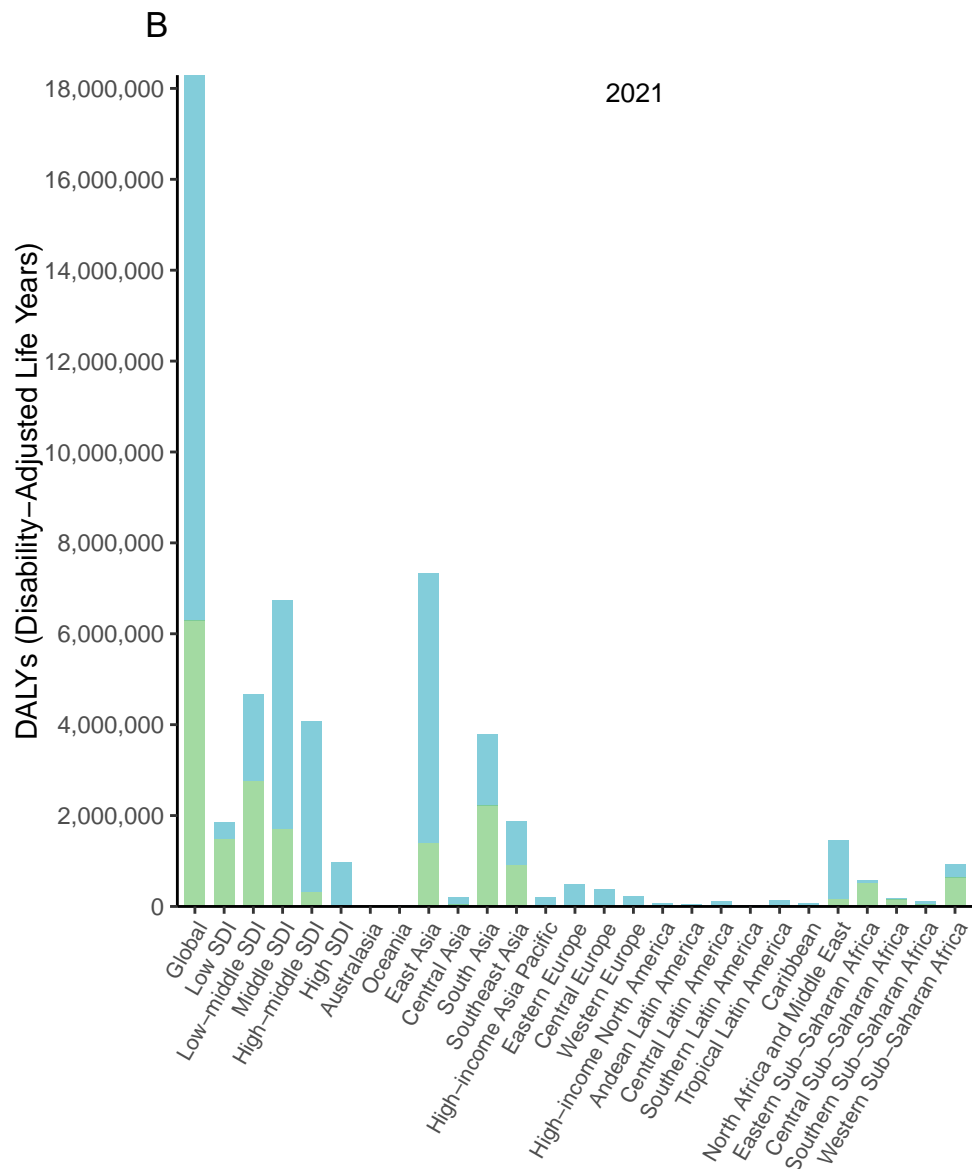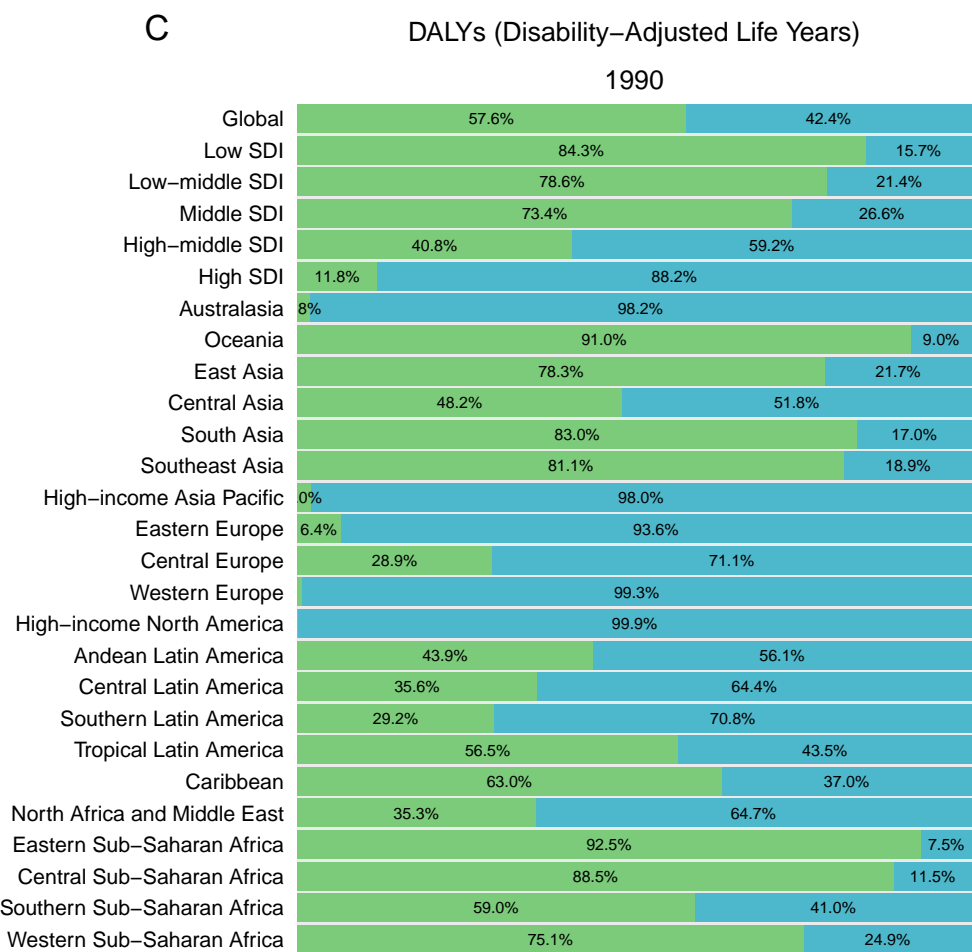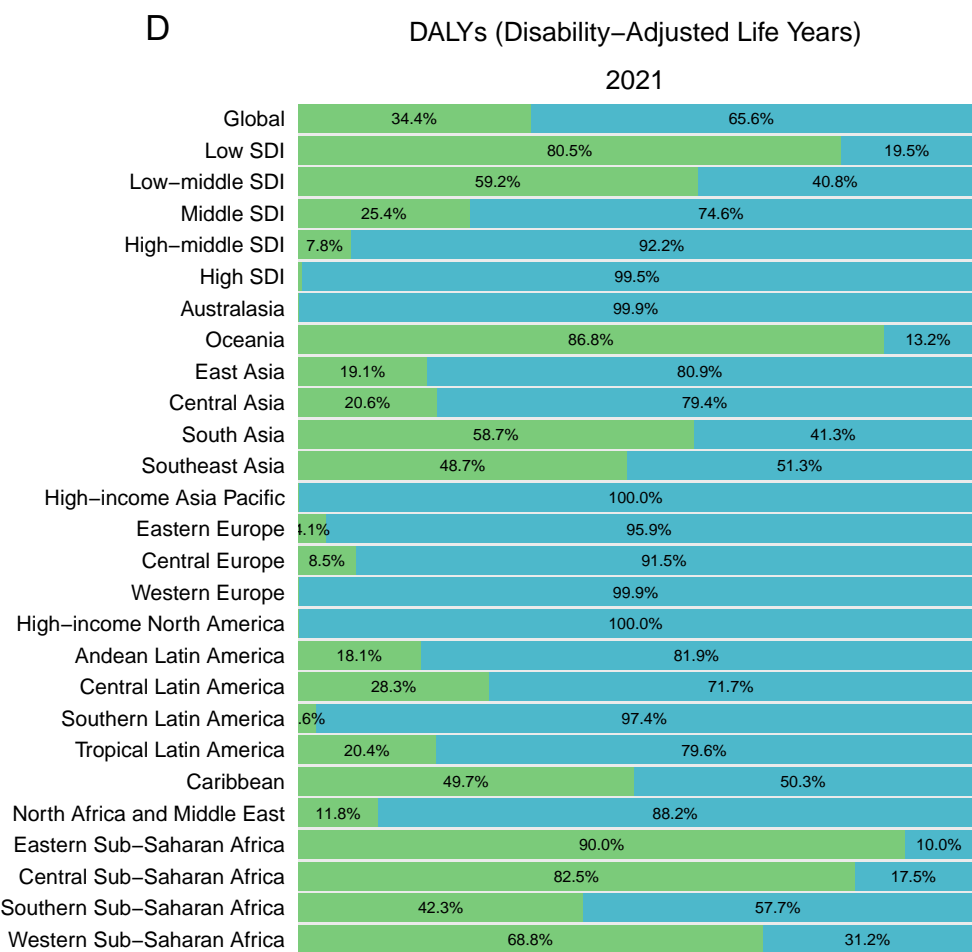

Supplement: SUPPLEMENTARY FIGURE S4 — Contribution of DALYs (numbers and percentages) from household and ambient pollution, globally and by region (1990-2021). [file Data_Sheet_4.PDF]

Ambient particulate matter pollution Household air pollution from solid fuels

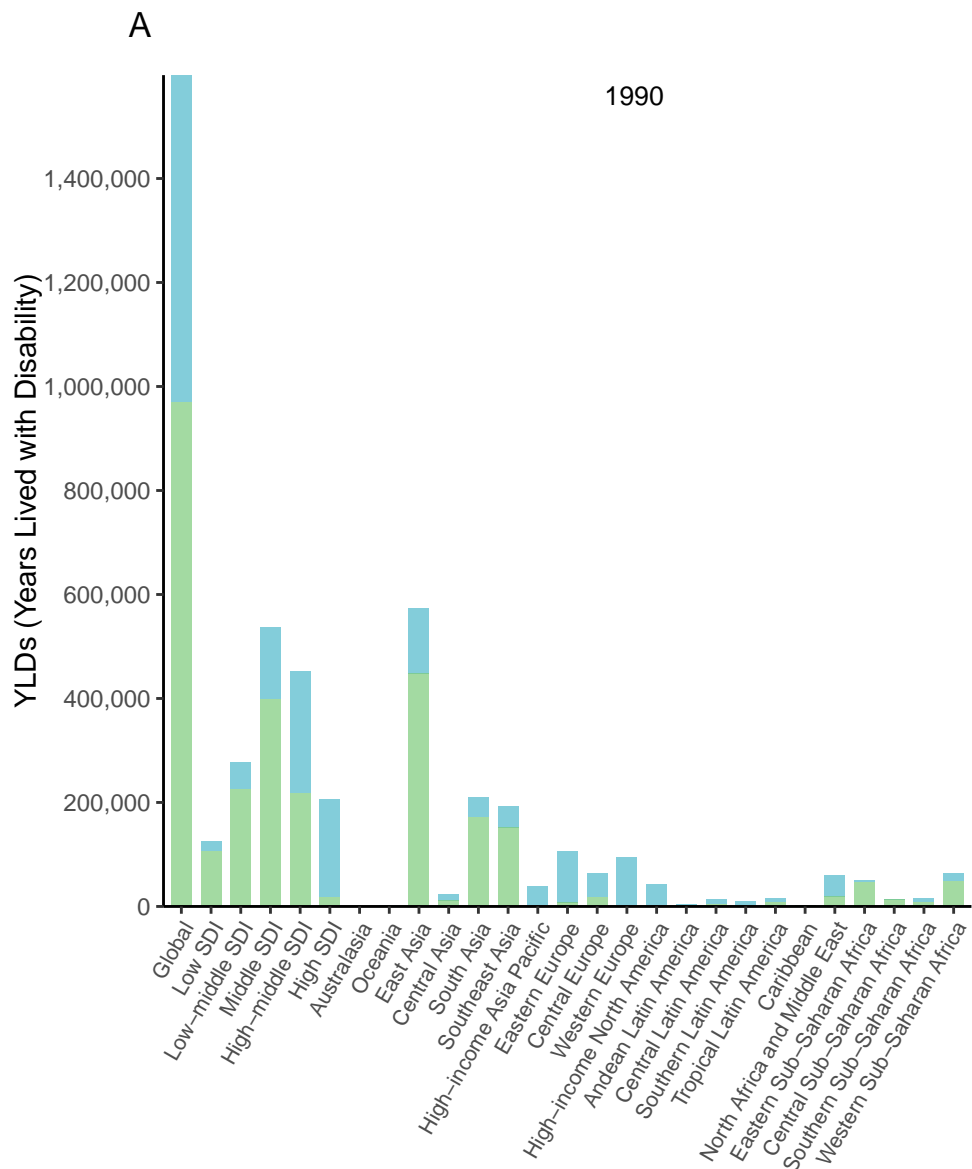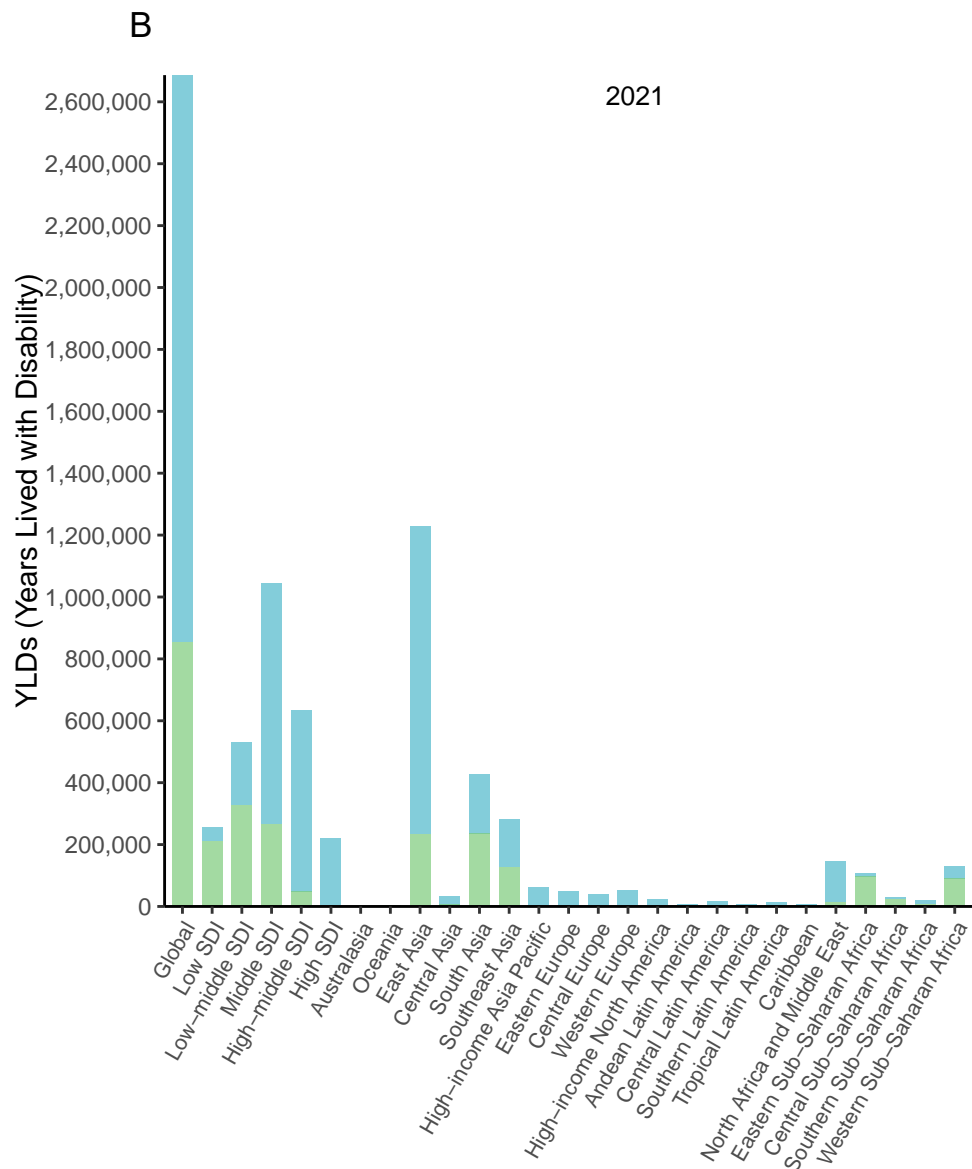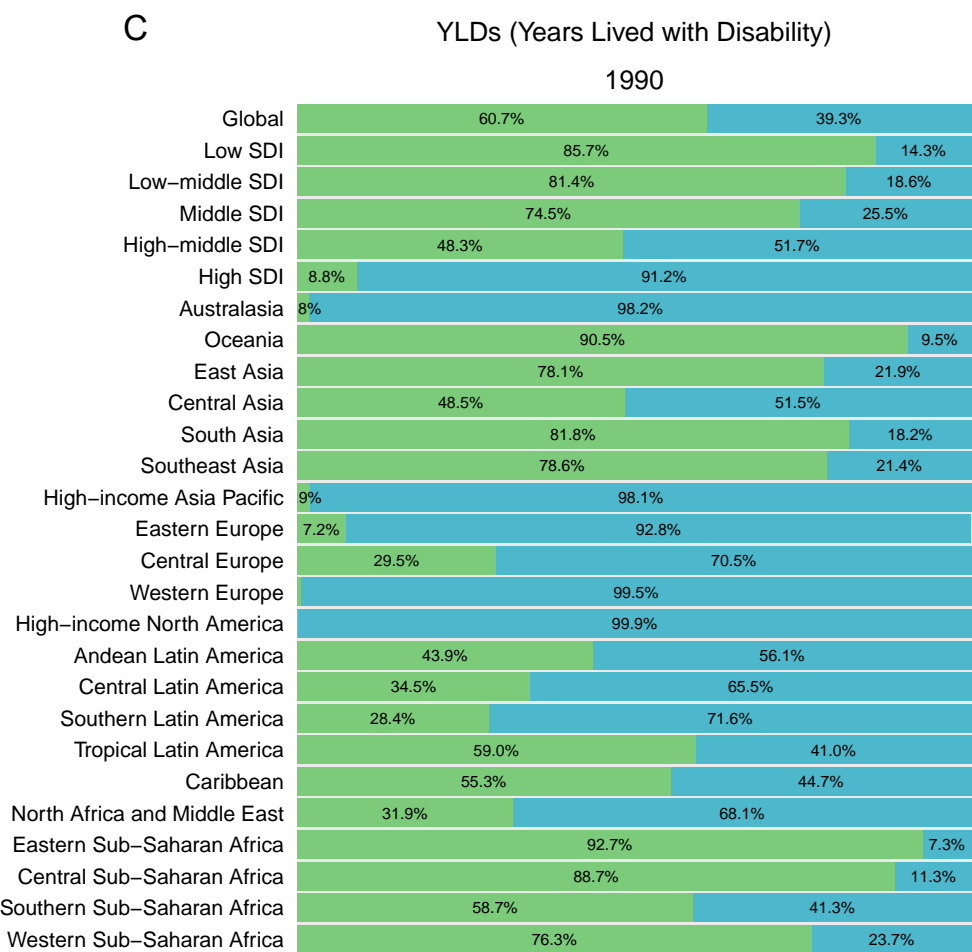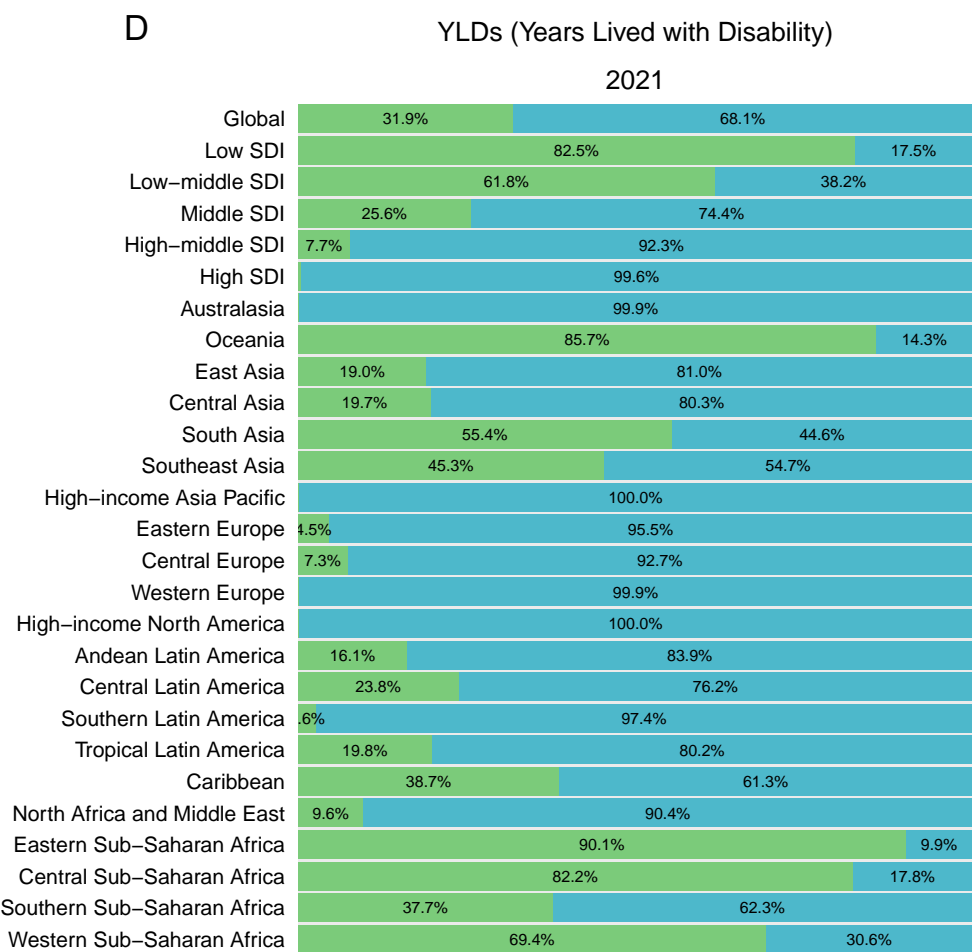

Supplement: SUPPLEMENTARY FIGURE S5 — Contribution of YLDs (numbers and percentages) from household and ambient pollution, globally and by region (1990-2021). [file Data_Sheet_5.PDF]

Ambient particulate matter pollution Household air pollution from solid fuels

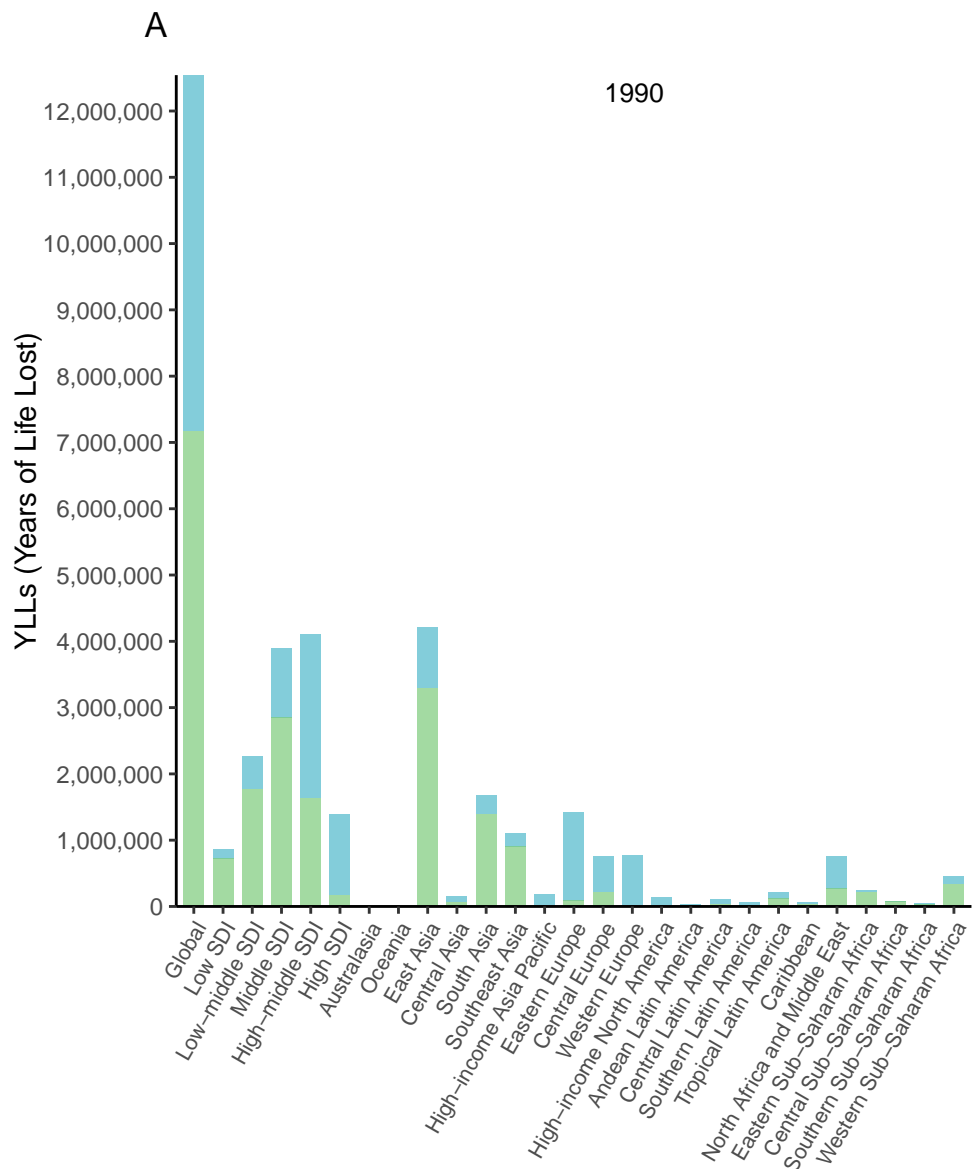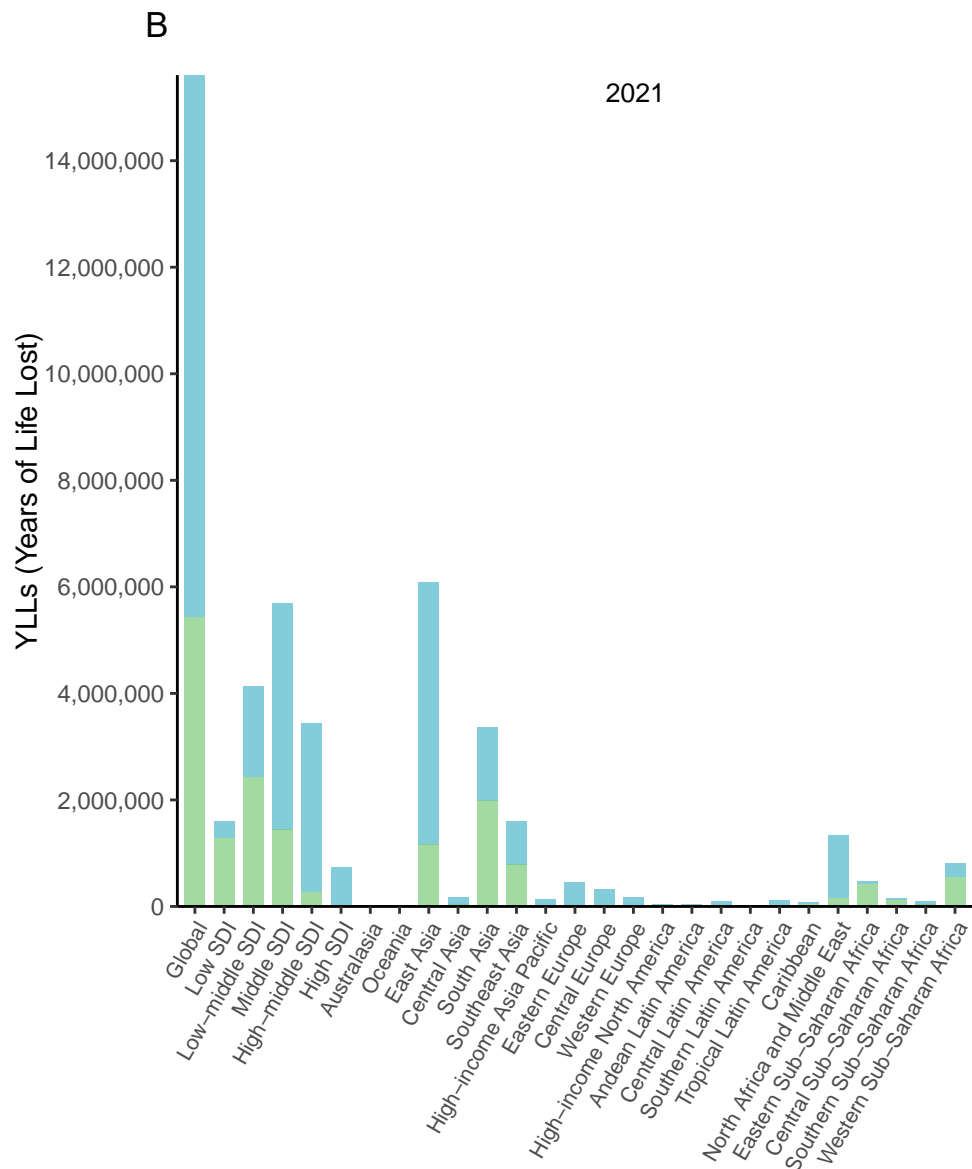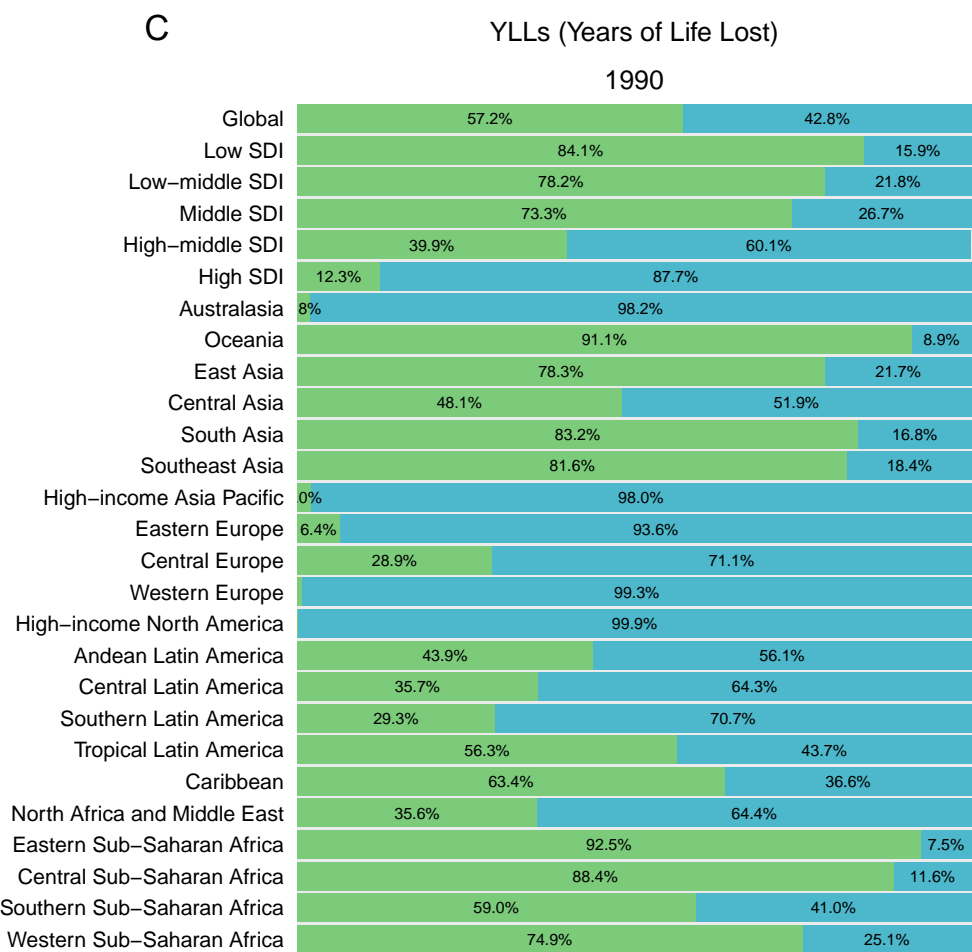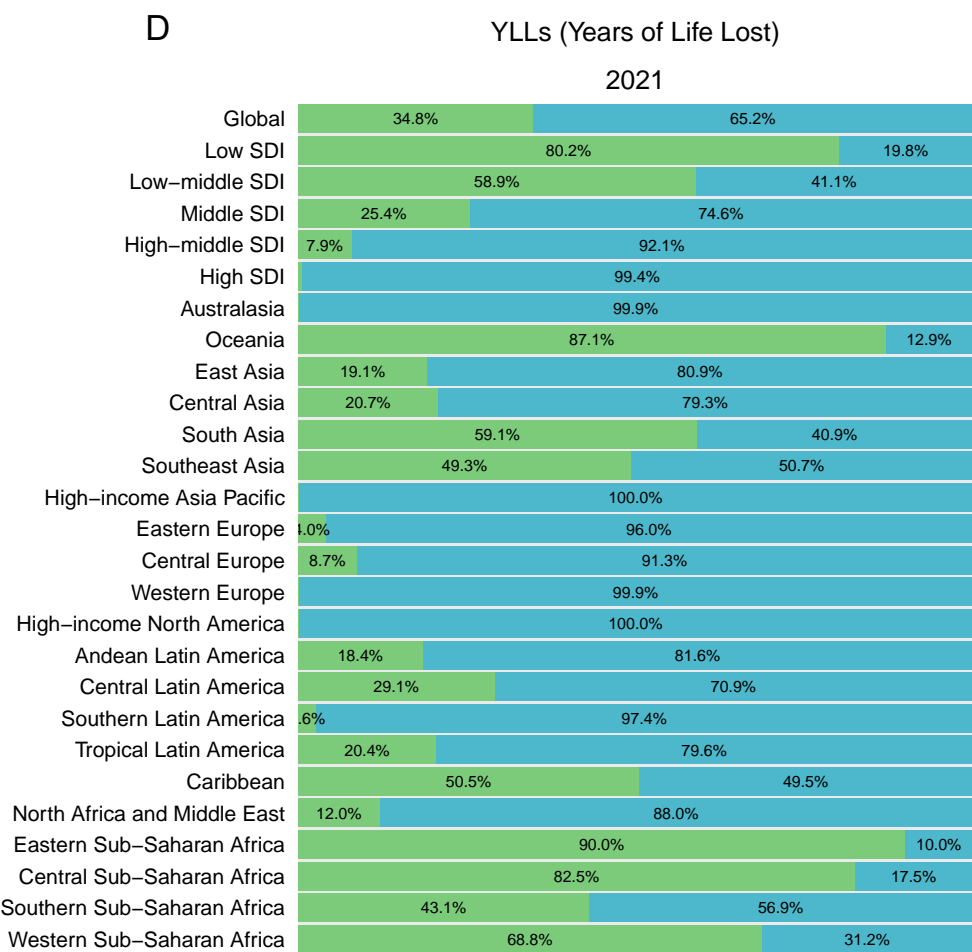

Supplement: SUPPLEMENTARY FIGURE S6 — Contribution of YLLs (numbers and percentages) from household and ambient pollution, globally and by region (1990-2021). [file Data_Sheet_6.PDF]

A

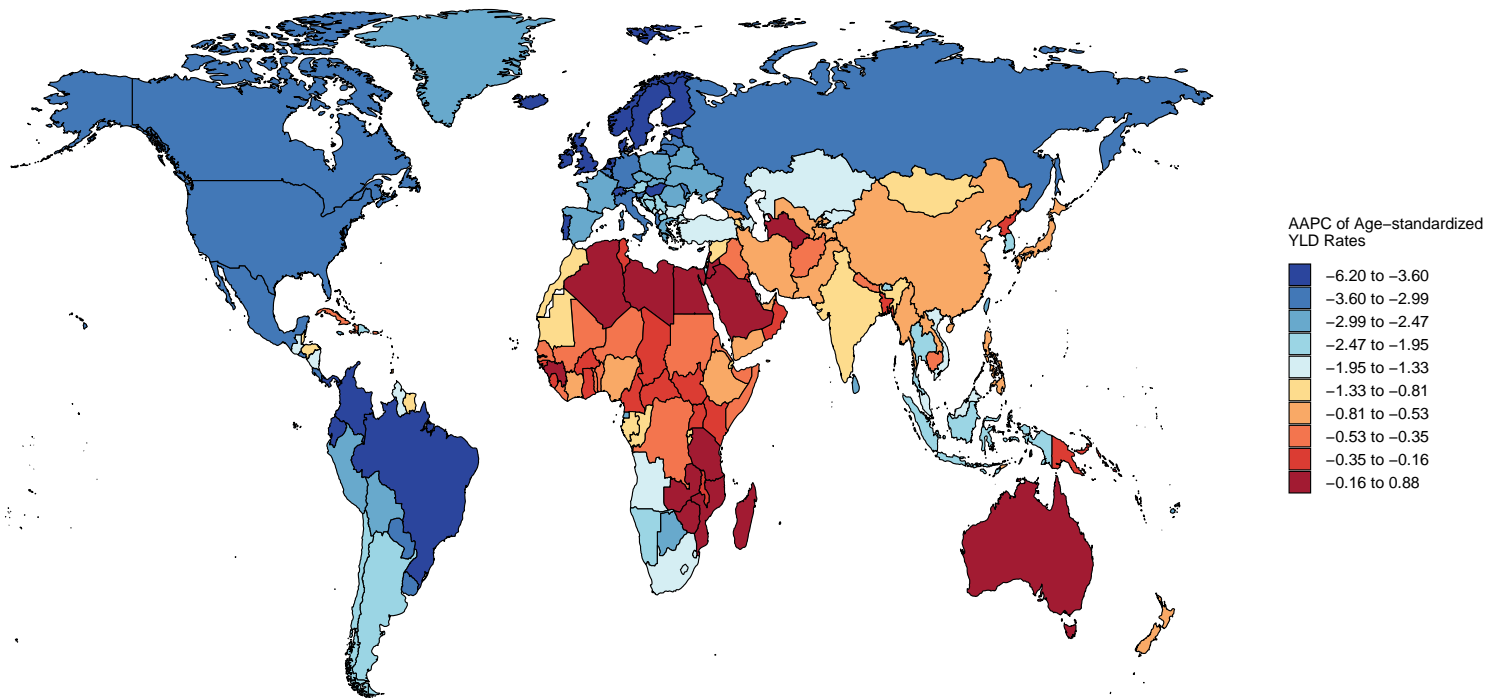

B

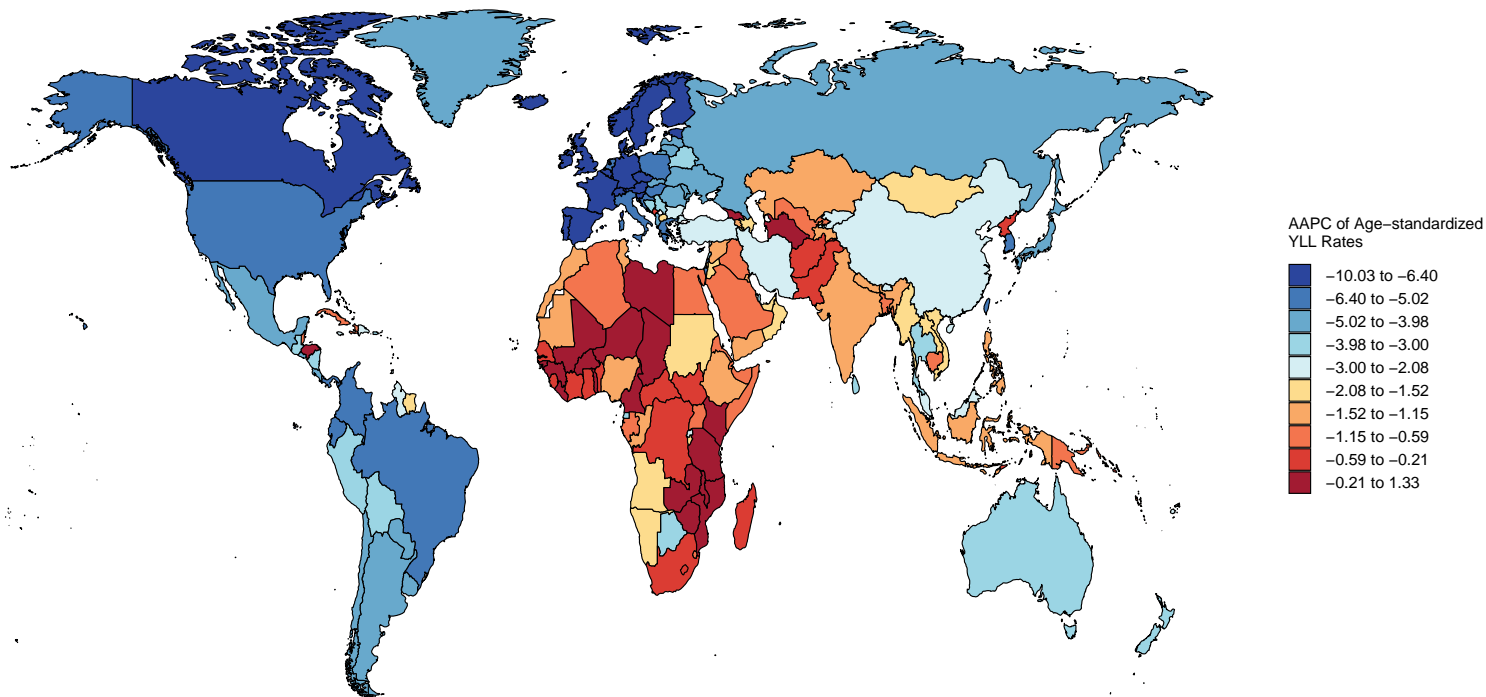

Supplement: SUPPLEMENTARY FIGURE S7 — Global distribution of the Average Annual Percent Change (AAPC) in age-standardized YLD (A) and YLL (B) rates from ischemic stroke linked to total pollution (1990-2021). [file Data_Sheet_7.PDF]

A

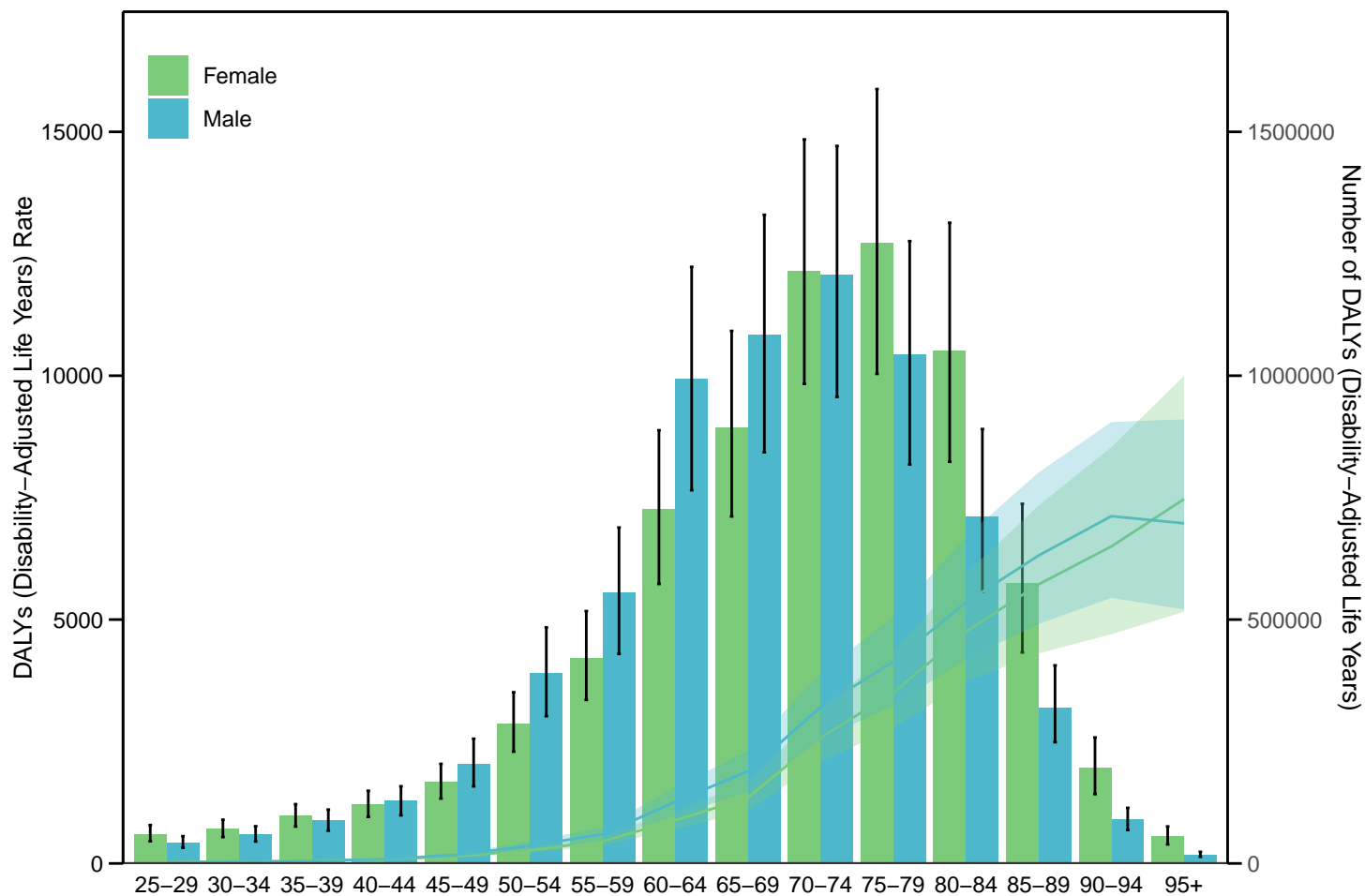

B

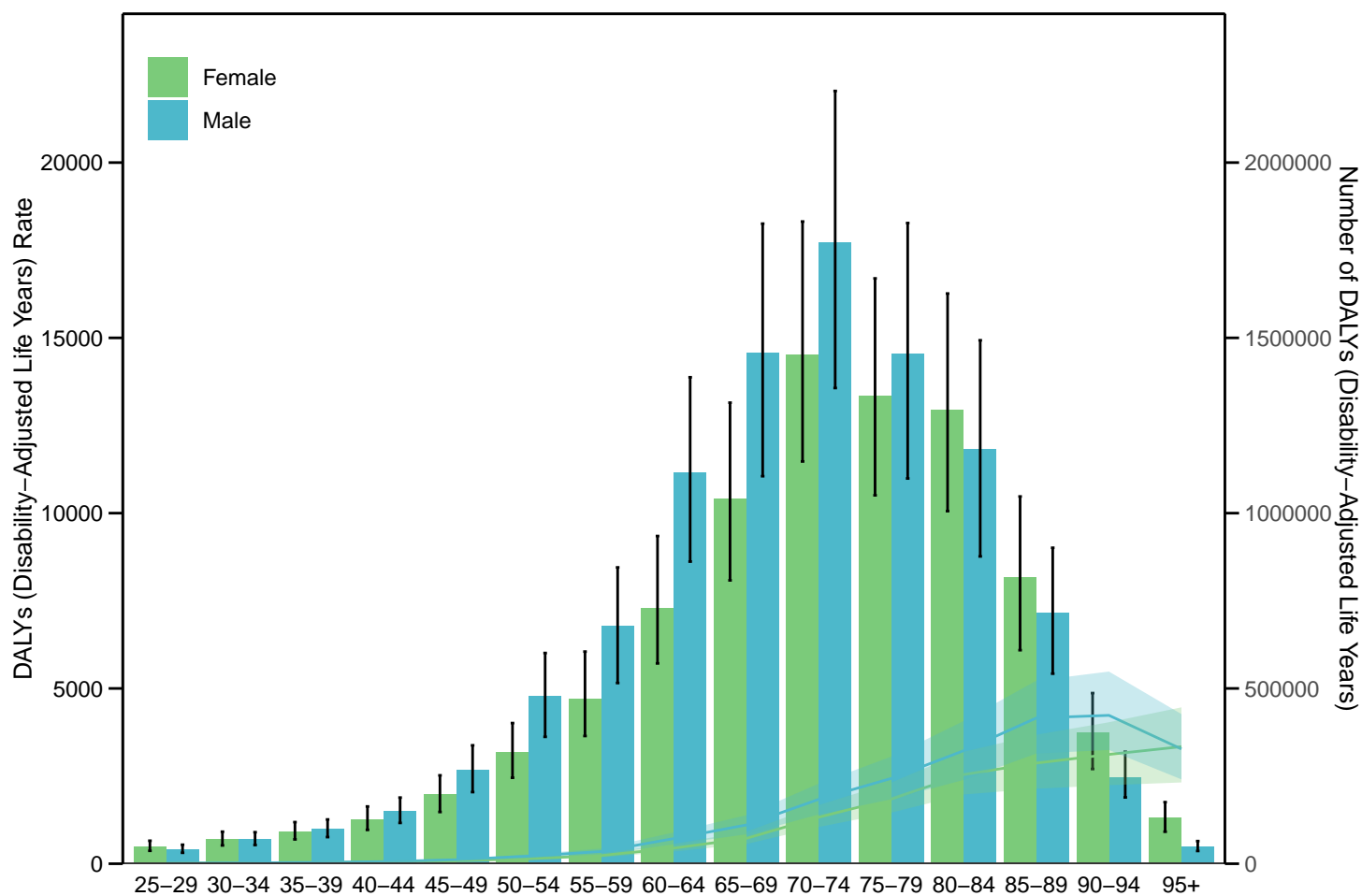

Supplement: SUPPLEMENTARY FIGURE S9 — Age-specific DALYs (bar chart) and age-adjusted DALYs rates (line chart) due to pollution in 1990 (A) and 2019 (B), by sex. [file Data_Sheet_9.PDF]

A

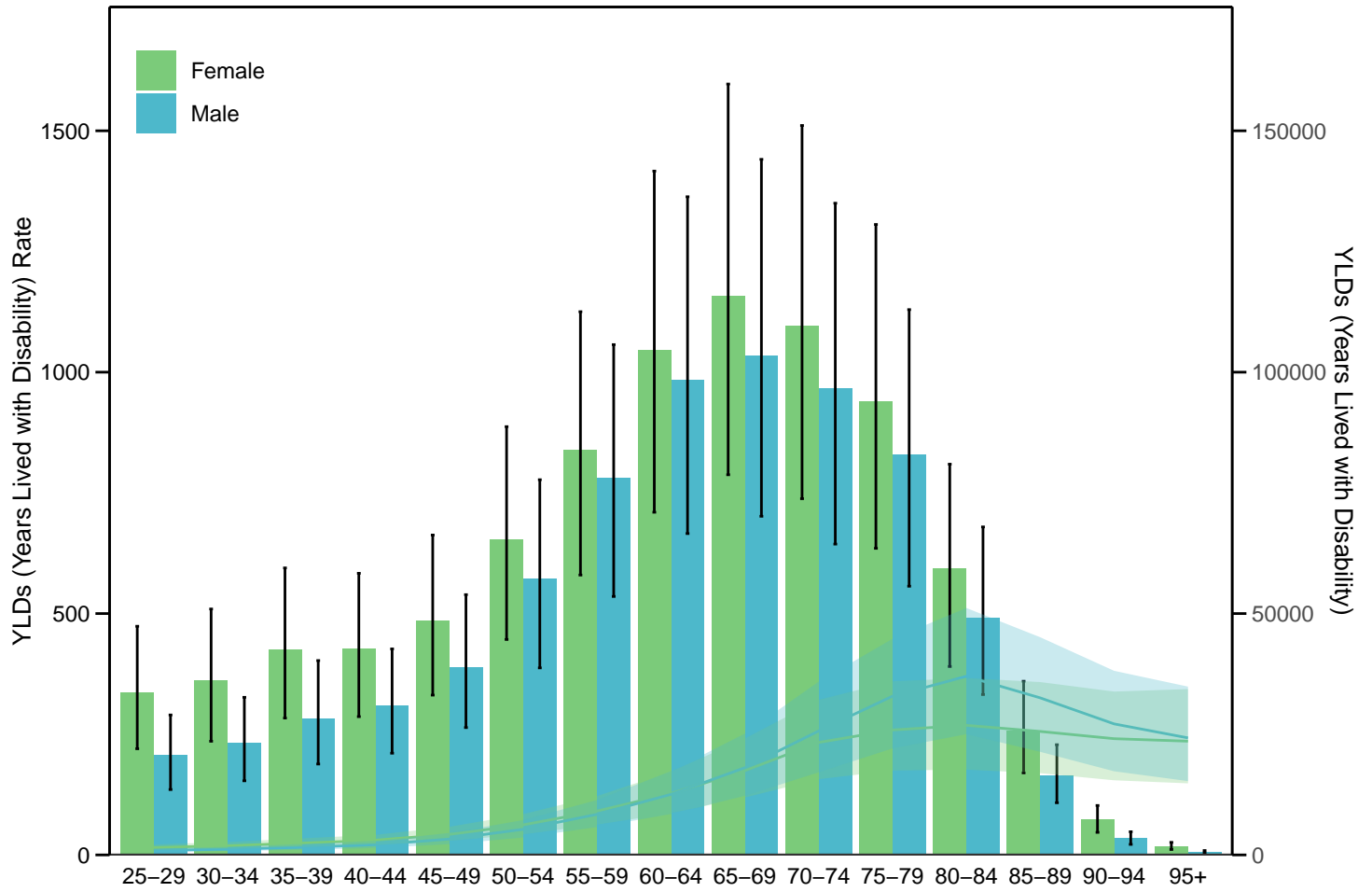

B

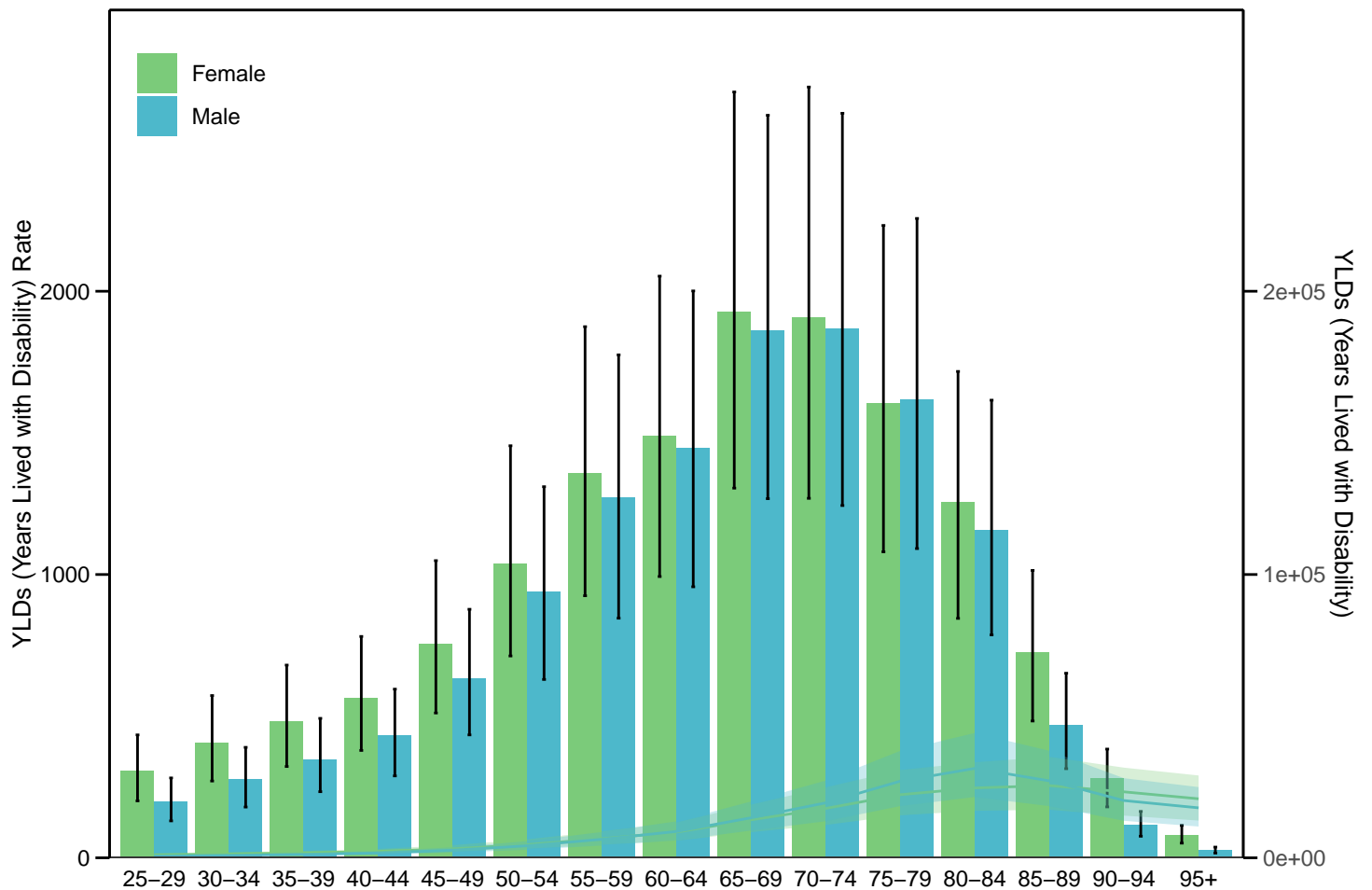

Supplement: SUPPLEMENTARY FIGURE S10 — Age-specific YLDs (bar chart) and age-adjusted YLDs rates (line chart) due to pollution in 1990 (A) and 2019 (B), by sex. [file Data_Sheet_10.PDF]

A

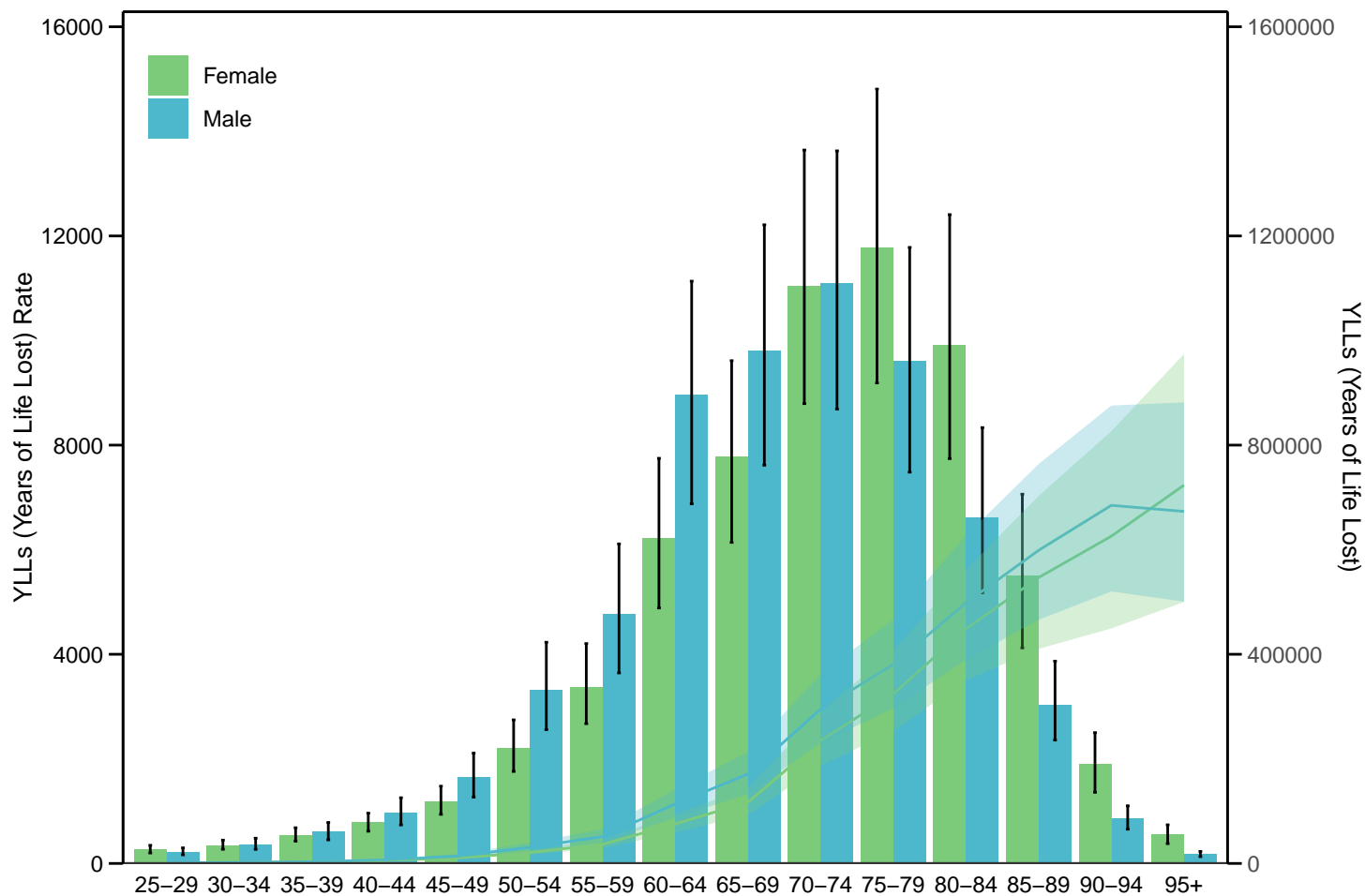

B

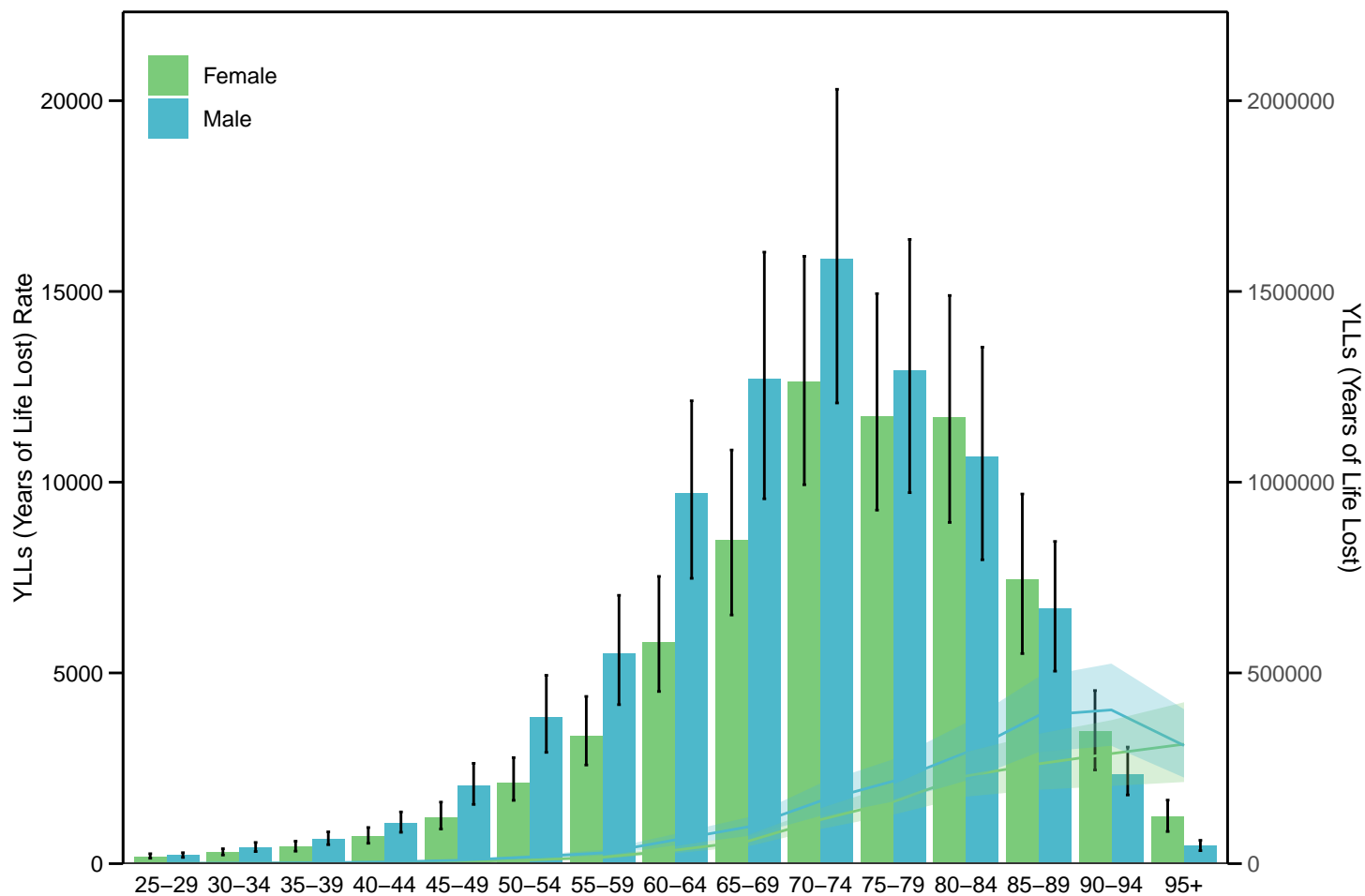

Supplement: SUPPLEMENTARY FIGURE S11 — Age-specific YLLs (bar chart) and age-adjusted YLLs rates (line chart) due to pollution in 1990 (A) and 2019 (B), by sex. [file Data_Sheet_11.PDF]
