# Supplementary material for: Global patterns and trends in ischemic stroke burden attributable to particulate matter pollution: changes from 1990 to 2021 and projections from 2022 to 2050
Source: Front Public Health. 2025 Jun 26;13:1599541. doi: 10.3389/fpubh.2025.1599541 (PMC12241019; doi:10.3389/fpubh.2025.1599541)
Supplement: Supplementary file 12 [file Table_1.docx]

| Table S1: Number and Age-Standardized DALYS Rates of Ischemic Stroke Attributable to Particulate Matter Pollution, with Temporal Trends from 1990 to 2021. | | | | | |
| --- | --- | --- | --- | --- | --- |
| Characteristics | **1990** | | **2021** | | **1990-2021** |
|  | **DALYs no.×10^3^**  **(95% UI)** | **Age-standardized DALYs rate per 100,000 (95% UI)** | **DALYs no.×10^3^**  **(95% UI)** | **Age-standardized DALYs rate per 100,000 (95% UI)** | **AAPC (95% CI)** |
| Global | 14141.35(11284.67-17056.67) | 385.24(306.72-467.72) | 18295.35(14324.97-22541.4) | 215.64(168.84-266.03) | -1.94(-2.29--1.58) |
| Female | 7206.97(5732.82-8882.57) | 351.11(279.21-434.2) | 8509.02(6686.1-10700.46) | 183.48(144.22-230.8) | -2.2(-2.39--2.01) |
| Male | 6934.38(5441.95-8480.03) | 427.43(334.03-522.6) | 9786.33(7459.37-12228.06) | 254.32(193.91-317.98) | -1.7(-1.86--1.54) |
| Low SDI | 989.09(773.34-1278.88) | 528.58(412.86-669.59) | 1852.56(1460.59-2348.45) | 433.63(345.01-543.78) | -0.64(-0.74--0.54) |
| Low-middle SDI | 2550.63(1999.8-3129.8) | 487.32(382.74-598.01) | 4658.82(3697.82-5706.83) | 358.32(285.59-437.76) | -1.08(-1.17--0.99) |
| Middle SDI | 4432.24(3560.4-5418.79) | 500.01(401.24-612.41) | 6742.53(4974.28-8716.82) | 270.23(199.58-349.64) | -2.05(-2.33--1.77) |
| High-middle SDI | 4555.41(3337.69-5815.57) | 499.61(364.15-642.44) | 4064.82(3025.09-5315.16) | 206.36(153.81-269.72) | -2.85(-3.26--2.44) |
| High SDI | 1595.02(1115.82-2231.18) | 141.77(98.81-198.11) | 963.04(722.06-1240.92) | 43.95(33.08-56.15) | -3.75(-4--3.5) |
| Australasia | 6.2(0.21-17.98) | 27.15(0.92-78.97) | 7.45(4.29-11.23) | 12.35(7.1-18.65) | -2.27(-2.98--1.56) |
| Oceania | 8.9(6.51-11.73) | 393.88(291.97-514.7) | 17.96(12.92-24.41) | 305.69(220.23-415.99) | -0.83(-0.88--0.77) |
| East Asia | 4783.94(3803.78-5943.22) | 648.02(510.41-804.86) | 7320.12(5352.99-9576.2) | 352.95(257.75-460.69) | -2(-2.35--1.65) |
| Central Asia | 176.49(100.96-260.46) | 400.79(229-591.62) | 212.73(157.64-272.8) | 290.6(214.85-373.6) | -1.12(-1.58--0.67) |
| South Asia | 1890.01(1444.49-2471.3) | 391.6(298.9-508.1) | 3794.47(2987.21-4997.88) | 285.73(225.61-369.84) | -1.04(-1.56--0.52) |
| Southeast Asia | 1302.29(1032.39-1552) | 603.05(477.48-717.64) | 1876.88(1290.4-2578.12) | 326.71(224.42-447.81) | -2.04(-2.27--1.81) |
| High-income Asia Pacific | 221.3(63.66-431.89) | 118.36(33.63-232.34) | 205.99(122.6-307.61) | 37.98(23.31-56.43) | -3.6(-3.87--3.34) |
| Eastern Europe | 1533.5(776.36-2384.88) | 583.72(295.95-908.41) | 504.11(314.99-759.91) | 140.97(88.08-212.43) | -4.49(-5.01--3.97) |
| Central Europe | 822.39(483.09-1165.71) | 591.05(348.08-836.17) | 368.34(271.35-532.39) | 155.8(114.61-225.77) | -4.28(-4.53--4.04) |
| Western Europe | 867.84(420.65-1455.66) | 142.17(68.89-238.44) | 223.83(151.46-312.24) | 19.86(13.37-27.67) | -6.18(-6.51--5.85) |
| High-income North America | 186.62(71.98-325.75) | 50.81(19.59-88.76) | 75.1(36.05-123.23) | 10.95(5.24-18) | -4.83(-5.32--4.34) |
| Andean Latin America | 44.91(34.07-56.31) | 238.81(181.26-299.33) | 37.73(24.46-54.24) | 66.09(42.88-95.13) | -4.07(-4.48--3.66) |
| Central Latin America | 128.28(84.1-178.81) | 174.28(113.97-242.26) | 112.38(76.17-159.84) | 47.05(31.9-66.95) | -4.13(-4.4--3.86) |
| Southern Latin America | 75.63(40.23-116.02) | 173.31(92.16-265.98) | 41.77(25.2-63.83) | 46.15(27.83-70.52) | -4.21(-4.47--3.96) |
| Tropical Latin America | 234.07(138.99-355.54) | 298.24(179.11-451) | 130.47(75.27-201.4) | 52.49(30.31-81.04) | -5.43(-5.62--5.23) |
| Caribbean | 58.16(40.34-82.14) | 234.39(161.76-331.84) | 80.17(54.1-111.01) | 148.84(100.51-206.04) | -1.4(-1.67--1.13) |
| North Africa and Middle East | 819.84(628.13-1029) | 557.79(426.67-699.27) | 1471.85(1110.77-1806.85) | 357.47(270.62-434.47) | -1.55(-1.68--1.42) |
| Eastern Sub-Saharan Africa | 291.48(228.06-374.32) | 485.63(382.45-617.3) | 576.26(459.37-715.17) | 422.04(334.5-519.73) | -0.45(-0.54--0.36) |
| Central Sub-Saharan Africa | 99.29(74.75-129.89) | 582.11(439-750.48) | 186.28(131.5-257.26) | 462.79(327.71-648.45) | -0.74(-0.9--0.59) |
| Southern Sub-Saharan Africa | 69.4(50.6-89.48) | 289.38(210.02-374.85) | 119.94(88.09-157.79) | 244.19(178.23-320.76) | -0.54(-0.96--0.11) |
| Western Sub-Saharan Africa | 520.81(395.35-689.85) | 689.21(525.51-901.26) | 931.54(718.73-1179.2) | 564.04(438.7-713.78) | -0.64(-0.72--0.56) |
