# Supplementary material for: Global patterns and trends in ischemic stroke burden attributable to particulate matter pollution: changes from 1990 to 2021 and projections from 2022 to 2050
Source: Front Public Health. 2025 Jun 26;13:1599541. doi: 10.3389/fpubh.2025.1599541 (PMC12241019; doi:10.3389/fpubh.2025.1599541)
Supplement: Supplementary file 13 [file Table_2.docx]

| Table S2: Number and Age-Standardized YLDs Rates of Ischemic Stroke Attributable to Particulate Matter Pollution, with Temporal Trends from 1990 to 2021. | | | | | |
| --- | --- | --- | --- | --- | --- |
| Characteristics | **1990** | | **2021** | | **1990-2021** |
|  | **YLDs no.×10^3^**  **(95% UI)** | **Age-standardized YLDs rate per 100,000 (95% UI)** | **YLDs no.×10^3^**  **(95% UI)** | **Age-standardized YLDs rate per 100,000 (95% UI)** | **AAPC (95% CI)** |
| Global | 1598.92(1118.23-2145.15) | 39.63(27.6-53.15) | 2686.69(1828.47-3692.88) | 31.27(21.26-42.95) | -0.82(-0.93--0.71) |
| Female | 870.65(608.85-1167.5) | 40.04(27.97-53.73) | 1419.84(968.35-1962.98) | 31.22(21.28-43.18) | -0.91(-1.15--0.67) |
| Male | 728.27(506-976.17) | 39.98(27.62-54.02) | 1266.85(860.12-1751.3) | 31.55(21.4-43.56) | -0.83(-0.95--0.7) |
| Low SDI | 125.26(88.77-166.41) | 55.29(39.48-72.83) | 255.89(181.33-337.38) | 48.86(34.88-64.06) | -0.41(-0.44--0.37) |
| Low-middle SDI | 277.45(193.64-372.95) | 44.07(30.99-58.74) | 530.83(368.99-722.12) | 35.89(25.05-48.55) | -0.74(-0.84--0.65) |
| Middle SDI | 536.78(368.7-728.59) | 51.34(35.71-69.55) | 1043.46(688.26-1479.86) | 39.42(25.97-55.75) | -1.04(-1.17--0.9) |
| High-middle SDI | 452.12(299.1-628.58) | 44.94(29.8-62.38) | 634.78(431.03-886.72) | 32.42(22.01-45.1) | -1.13(-1.23--1.04) |
| High SDI | 205.66(123.35-314.93) | 18.66(11.24-28.49) | 220.13(138.69-312.74) | 11.03(7.02-15.74) | -1.7(-1.77--1.63) |
| Australasia | 0.95(0.02-2.61) | 4.02(0.1-11.09) | 2.22(1.21-3.45) | 4.16(2.26-6.47) | 0.04(-1.41-1.52) |
| Oceania | 1.73(1.2-2.3) | 59.9(41.57-79.95) | 3.66(2.49-4.96) | 48.69(33.19-66.22) | -0.68(-0.7--0.65) |
| East Asia | 573.88(389.9-784.91) | 67.17(45.89-91.53) | 1228.32(824.47-1731.63) | 57.27(38.42-80.76) | -0.61(-0.74--0.48) |
| Central Asia | 23.8(13.14-37.29) | 49.27(27.1-77.48) | 33.8(21.59-46.67) | 39.95(25.51-55.02) | -0.74(-0.82--0.66) |
| South Asia | 209.88(145.15-283.09) | 34.78(23.99-46.7) | 427.78(300.47-586.56) | 28.09(19.66-38.16) | -0.78(-1--0.56) |
| Southeast Asia | 193.35(130.63-267.93) | 73.89(50.51-101.72) | 281.14(179.48-413.42) | 42.83(27.16-63.07) | -1.83(-1.96--1.7) |
| High-income Asia Pacific | 38.19(10.49-76.23) | 19.11(5.22-38.26) | 61.97(33.83-95.34) | 14.13(7.9-21.59) | -0.97(-1.09--0.86) |
| Eastern Europe | 105.95(52.08-177.19) | 37.63(18.52-62.85) | 48.75(27.82-74.99) | 14.37(8.19-22.09) | -3.23(-3.51--2.95) |
| Central Europe | 62.8(34.83-95.77) | 42.36(23.54-64.58) | 39.76(25.99-57.44) | 18.43(12.1-26.67) | -2.68(-2.8--2.55) |
| Western Europe | 93.72(43.43-165.28) | 16.09(7.46-28.32) | 52.57(32.56-77.75) | 5.54(3.41-8.22) | -3.43(-3.5--3.36) |
| High-income North America | 43.18(15.1-81.06) | 12.23(4.27-22.92) | 24.44(11.05-43.01) | 3.9(1.76-6.87) | -3.47(-3.89--3.06) |
| Andean Latin America | 4.76(3.23-6.66) | 22.72(15.46-31.64) | 5.58(3.51-8.06) | 9.37(5.9-13.51) | -2.97(-3.2--2.73) |
| Central Latin America | 14.62(8.47-22.24) | 17.29(9.98-26.36) | 16.02(9.98-23.96) | 6.46(4.03-9.66) | -3.11(-3.17--3.05) |
| Southern Latin America | 9.86(4.93-16.49) | 21.51(10.72-35.98) | 9.31(5-15.08) | 10.72(5.75-17.36) | -2.24(-2.46--2.02) |
| Tropical Latin America | 14.53(8.28-22.85) | 16.02(9.24-25.26) | 13.29(7.05-21.95) | 5.23(2.77-8.68) | -3.6(-3.69--3.5) |
| Caribbean | 3.41(2.09-5.53) | 12.81(7.89-20.82) | 5.37(3.23-8.16) | 10.07(6.07-15.28) | -0.75(-0.89--0.61) |
| North Africa and Middle East | 59.68(41.92-81.66) | 32.11(22.53-43.89) | 144.66(99.81-192.57) | 29.03(20.13-38.94) | -0.41(-0.54--0.27) |
| Eastern Sub-Saharan Africa | 50.82(35.98-66.52) | 70.58(50.15-91.79) | 108.08(76.87-140.09) | 64.9(46.31-83.74) | -0.27(-0.28--0.26) |
| Central Sub-Saharan Africa | 15.16(10.8-19.93) | 71.12(50.83-92.38) | 30.33(21.48-39.86) | 57.16(40.79-75.28) | -0.71(-0.73--0.69) |
| Southern Sub-Saharan Africa | 14.71(9.8-20.6) | 55.64(36.97-78.4) | 18.79(12.38-26.4) | 34.22(22.32-48.24) | -1.55(-1.66--1.45) |
| Western Sub-Saharan Africa | 63.96(45.56-84.79) | 72.1(51.17-95.11) | 130.87(91.99-171.4) | 63.52(44.89-83.17) | -0.41(-0.44--0.38) |
