# Supplementary material for: Global patterns and trends in ischemic stroke burden attributable to particulate matter pollution: changes from 1990 to 2021 and projections from 2022 to 2050
Source: Front Public Health. 2025 Jun 26;13:1599541. doi: 10.3389/fpubh.2025.1599541 (PMC12241019; doi:10.3389/fpubh.2025.1599541)
Supplement: Supplementary file 14 [file Table_3.docx]

| Table S3: Number and Age-Standardized YLLs Rates of Ischemic Stroke Attributable to Particulate Matter Pollution, with Temporal Trends from 1990 to 2021. | | | | | |
| --- | --- | --- | --- | --- | --- |
| Characteristics | **1990** | | **2021** | | **1990-2021** |
|  | **YLLs no.×10^3^**  **(95% UI)** | **Age-standardized YLLs rate per 100,000 (95% UI)** | **YLLs no.×10^3^**  **(95% UI)** | **Age-standardized YLLs rate per 100,000 (95% UI)** | **AAPC (95% CI)** |
| Global | 12542.43(9920.37-15259.97) | 345.61(273.11-422.26) | 15608.66(12088.47-19455.22) | 184.37(142.74-230.24) | -2.05(-2.22--1.87) |
| Female | 6336.32(5015.04-7917.41) | 311.06(246.26-389.89) | 7089.18(5546.95-8974.7) | 152.26(119.17-192.73) | -2.41(-2.6--2.21) |
| Male | 6206.11(4825.77-7638.14) | 387.45(301.1-476.75) | 8519.48(6473.42-10732.46) | 222.77(169.38-281.35) | -1.81(-1.99--1.62) |
| Low SDI | 863.83(658.18-1147.87) | 473.29(365.22-618.68) | 1596.67(1242.25-2097.38) | 384.76(302.5-494.08) | -0.66(-0.78--0.55) |
| Low-middle SDI | 2273.18(1769.89-2838.99) | 443.25(344.37-548.87) | 4127.98(3261.34-5117.86) | 322.42(256.47-399.28) | -1.12(-1.21--1.03) |
| Middle SDI | 3895.46(3106.56-4814.22) | 448.68(356.43-554) | 5699.07(4208.31-7463.59) | 230.81(170.41-302.74) | -2.21(-2.51--1.9) |
| High-middle SDI | 4103.29(3004.64-5281.93) | 454.67(332.88-589.31) | 3430.05(2538-4532.29) | 173.94(128.74-229.76) | -3.09(-3.54--2.62) |
| High SDI | 1389.36(955.36-1947.47) | 123.11(84.35-172.47) | 742.91(561.57-965.01) | 32.92(25.23-42.4) | -4.22(-4.51--3.94) |
| Australasia | 5.25(0.18-15.36) | 23.13(0.81-67.78) | 5.23(2.98-7.93) | 8.2(4.68-12.41) | -3.15(-3.88--2.41) |
| Oceania | 7.17(5.03-9.75) | 333.98(238.17-444.17) | 14.3(10.1-20.34) | 256.99(182.83-359.4) | -0.85(-0.91--0.8) |
| East Asia | 4210.06(3284.02-5316.79) | 580.85(454.52-732.41) | 6091.79(4391.92-8086.09) | 295.68(214.18-391.35) | -2.21(-2.52--1.91) |
| Central Asia | 152.69(87.47-227.17) | 351.52(200.39-522.67) | 178.93(132.93-234.97) | 250.65(185.22-329.03) | -1.18(-1.67--0.69) |
| South Asia | 1680.13(1255.27-2245.28) | 356.82(269.53-468.22) | 3366.69(2610.91-4487.95) | 257.64(200.51-338.81) | -1.07(-1.63--0.51) |
| Southeast Asia | 1108.94(853.12-1356.37) | 529.16(408.68-643.73) | 1595.75(1066.64-2216.23) | 283.88(191.43-391.71) | -2.07(-2.31--1.83) |
| High-income Asia Pacific | 183.11(53.02-368.2) | 99.24(28.1-200.59) | 144.02(85.36-213.91) | 23.85(14.14-34.99) | -4.53(-4.89--4.17) |
| Eastern Europe | 1427.55(717.3-2220.94) | 546.08(273.06-848.58) | 455.36(285.53-688.9) | 126.6(79.37-191.48) | -4.61(-5.15--4.07) |
| Central Europe | 759.59(447.49-1065.62) | 548.69(323.11-769.08) | 328.58(242.04-470.15) | 137.37(101.09-196.79) | -4.44(-4.71--4.18) |
| Western Europe | 774.12(374.59-1277.48) | 126.07(61.03-207.96) | 171.25(115.24-243.96) | 14.32(9.65-20.43) | -6.81(-7.15--6.47) |
| High-income North America | 143.45(55.53-255.01) | 38.58(14.94-68.59) | 50.66(24.99-84.02) | 7.05(3.47-11.64) | -5.29(-5.78--4.81) |
| Andean Latin America | 40.16(30.24-50.41) | 216.09(162.95-271.22) | 32.15(20.67-46.6) | 56.72(36.55-82.24) | -4.22(-4.68--3.76) |
| Central Latin America | 113.66(74.13-156.06) | 156.98(102.38-215.72) | 96.36(65.49-138.24) | 40.59(27.57-58.3) | -4.26(-4.56--3.96) |
| Southern Latin America | 65.76(35.62-99.75) | 151.8(82.21-230.61) | 32.46(19.71-49.33) | 35.43(21.53-53.86) | -4.65(-4.91--4.4) |
| Tropical Latin America | 219.54(129.09-332.39) | 282.22(167.88-423.38) | 117.18(68.12-179.92) | 47.26(27.48-72.56) | -5.57(-5.77--5.37) |
| Caribbean | 54.75(37.7-77.95) | 221.58(152.01-315.98) | 74.81(50.98-104.04) | 138.78(94.69-193.07) | -1.45(-1.73--1.16) |
| North Africa and Middle East | 760.16(583.96-962.38) | 525.67(400.44-662.58) | 1327.19(995.15-1639.07) | 328.44(246.78-402.3) | -1.63(-1.76--1.5) |
| Eastern Sub-Saharan Africa | 240.66(180.95-316.44) | 415.04(318.04-541.84) | 468.18(361.75-593.21) | 357.15(276.9-446.26) | -0.48(-0.58--0.39) |
| Central Sub-Saharan Africa | 84.13(60.58-112.75) | 510.99(372.51-669.28) | 155.95(104.22-222.95) | 405.63(275.47-588.91) | -0.77(-0.9--0.63) |
| Southern Sub-Saharan Africa | 54.69(39.01-70.71) | 233.74(167.1-303.5) | 101.15(73.26-131.88) | 209.98(152.35-275) | -0.32(-0.8-0.15) |
| Western Sub-Saharan Africa | 456.85(334.83-611.99) | 617.11(455.76-821.83) | 800.67(605.87-1023.01) | 500.52(381.26-639.32) | -0.67(-0.76--0.58) |
