# Supplementary material for: Global patterns and trends in ischemic stroke burden attributable to particulate matter pollution: changes from 1990 to 2021 and projections from 2022 to 2050
Source: Front Public Health. 2025 Jun 26;13:1599541. doi: 10.3389/fpubh.2025.1599541 (PMC12241019; doi:10.3389/fpubh.2025.1599541)
Supplement: SUPPLEMENTARY FIGURE S8 — Correlation between age-standardized YLLs and YLDs for ischemic stroke due to pollution and the Socio-demographic Index across 204 countries in 2021. [file Data_Sheet_8.PDF]

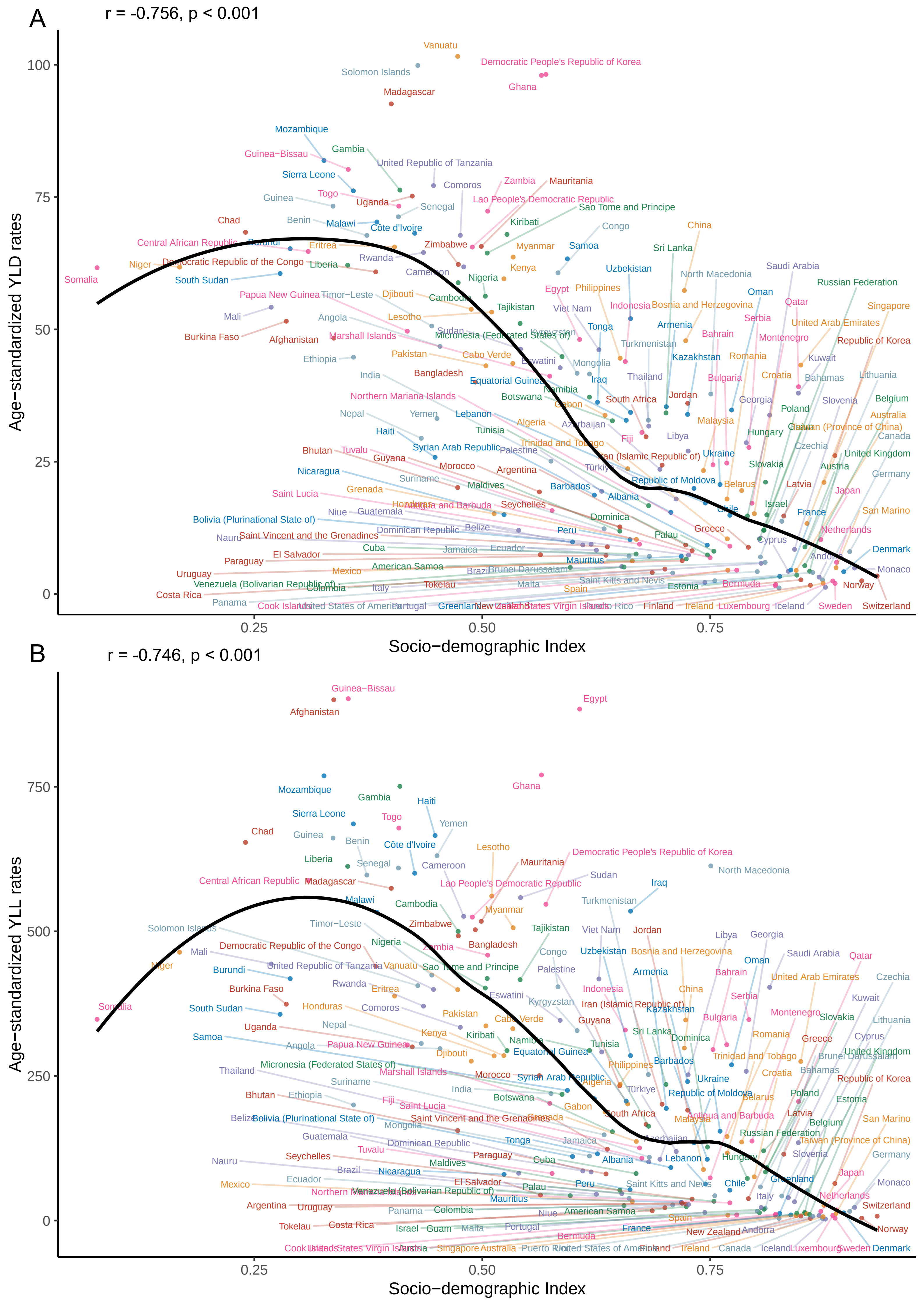
A
r = -0.756, p < 0.001
Age−standardized YLD rates
Socio−demographic Index
0
25
50
75
100
0.25
0.50
0.75
Vanuatu
Solomon Islands
Democratic People's Republic of Korea
Ghana
Madagascar
Mozambique
Gambia
Guinea−Bissau
United Republic of Tanzania
Sierra Leone
Zambia
Mauritania
Comoros
Togo
Guinea
Uganda
Senegal
Lao People's Democratic Republic
Sao Tome and Principe
Chad
Benin
Malawi
Kiribati
Côte d'Ivoire
Congo
China
Central African Republic
Burundi
Eritrea
Zimbabwe
Myanmar
Samoa
Sri Lanka
Niger
Democratic Republic of the Congo
Liberia
Rwanda
Kenya
Uzbekistan
Saudi Arabia
Somalia
South Sudan
Cameroon
Nigeria
North Macedonia
Russian Federation
Papua New Guinea
Timor−Leste
Djibouti
Cambodia
Egypt
Philippines
Oman
Tajikistan
Viet Nam
Indonesia
Bosnia and Herzegovina
Qatar
Singapore
Mali
Lesotho
Angola
Tonga
Armenia
Serbia
Burkina Faso
Afghanistan
Marshall Islands
Sudan
Micronesia (Federated States of)
Kyrgyzstan
Turkmenistan
Bahrain
United Arab Emirates
Montenegro
Republic of Korea
Ethiopia
Pakistan
Cabo Verde
Eswatini
Mongolia
Kazakhstan
Romania
Kuwait
India
Bangladesh
Equatorial Guinea
Iraq
Thailand
Bulgaria
Croatia
Bahamas
Lithuania
Northern Mariana Islands
Namibia
Botswana
Gabon
South Africa
Jordan
Georgia
Slovenia
Belgium
Nepal
Yemen
Lebanon
Poland
Australia
Haiti
Tunisia
Algeria
Azerbaijan
Malaysia
Hungary
Taiwan (Province of China)
Canada
Fiji
Libya
Syrian Arab Republic
Palestine
Trinidad and Tobago
Czechia
Bhutan
Tuvalu
Guyana
Iran (Islamic Republic of)
Ukraine
United Kingdom
Morocco
Türkiye
Slovakia
Austria
Nicaragua
Argentina
Suriname
Republic of Moldova
Germany
Maldives
Barbados
Belarus
Latvia
Saint Lucia
Grenada
Japan
Albania
Israel
Honduras
Antigua and Barbuda
Seychelles
Chile
France
San Marino
Niue
Guatemala
Bolivia (Plurinational State of)
Dominica
Greece
Netherlands
Dominican Republic
Belize
Peru
Nauru
Saint Vincent and the Grenadines
Palau
Cyprus
Cuba
Jamaica
Ecuador
Denmark
El Salvador
Andorra
Paraguay
Mauritius
American Samoa
Brazil
Brunei Darussalam
Monaco
Uruguay
Mexico
Saint Kitts and Nevis
Venezuela (Bolivarian Republic of)
Tokelau
Malta
Estonia
Bermuda
Norway
Colombia
Italy
Spain
Costa Rica
Panama
Cook Islands
United States of America
Portugal
Greenland
New Zealand
United States Virgin Islands
Puerto Rico
Finland
Ireland
Luxembourg
Iceland
Sweden
Switzerland
B
r = -0.746, p < 0.001
Age−standardized YLL rates
Socio−demographic Index
0
250
500
750
0.25
0.50
0.75
Guinea−Bissau
Egypt
Afghanistan
Mozambique
Ghana
Gambia
Haiti
Sierra Leone
Togo
Yemen
Chad
Guinea
Benin
Côte d'Ivoire
Lesotho
Democratic People's Republic of Korea
Liberia
Senegal
Mauritania
Cameroon
North Macedonia
Sudan
Central African Republic
Madagascar
Lao People's Democratic Republic
Iraq
Malawi
Cambodia
Turkmenistan
Myanmar
Timor−Leste
Zimbabwe
Tajikistan
Viet Nam
Jordan
Libya
Georgia
Solomon Islands
Nigeria
Bangladesh
Democratic Republic of the Congo
Zambia
Congo
Uzbekistan
Bosnia and Herzegovina
Saudi Arabia
Qatar
Mali
Niger
United Republic of Tanzania
Vanuatu
Sao Tome and Principe
Palestine
Oman
Burundi
Armenia
Bahrain
United Arab Emirates
Czechia
Rwanda
Eritrea
Eswatini
Indonesia
China
Burkina Faso
Kyrgyzstan
Iran (Islamic Republic of)
Serbia
Kuwait
Somalia
Honduras
Comoros
Kazakhstan
Montenegro
South Sudan
Pakistan
Cabo Verde
Bulgaria
Slovakia
Lithuania
Uganda
Nepal
Guyana
Samoa
Kenya
Kiribati
Sri Lanka
Romania
Cyprus
Namibia
Dominica
Greece
Angola
Papua New Guinea
Tunisia
Equatorial Guinea
Djibouti
United Kingdom
Trinidad and Tobago
Brunei Darussalam
Micronesia (Federated States of)
Philippines
Barbados
Thailand
Marshall Islands
Morocco
Syrian Arab Republic
Croatia
Bahamas
Suriname
Algeria
Ukraine
Republic of Korea
India
Türkiye
Bhutan
Ethiopia
Botswana
Republic of Moldova
Poland
Fiji
Belarus
Estonia
Saint Lucia
Gabon
South Africa
Belize
Bolivia (Plurinational State of)
Saint Vincent and the Grenadines
Grenada
Antigua and Barbuda
Latvia
Mongolia
Malaysia
Belgium
San Marino
Guatemala
Tonga
Jamaica
Azerbaijan
Russian Federation
Taiwan (Province of China)
Dominican Republic
Tuvalu
Paraguay
Nauru
Seychelles
Cuba
Albania
Lebanon
Hungary
Slovenia
Germany
Maldives
Brazil
Nicaragua
Greenland
Japan
Ecuador
El Salvador
Peru
Saint Kitts and Nevis
Chile
Monaco
Mexico
Northern Mariana Islands
Venezuela (Bolivarian Republic of)
Palau
Italy
Mauritius
Netherlands
Argentina
Uruguay
Panama
Colombia
Niue
American Samoa
Spain
Switzerland
Tokelau
Costa Rica
Israel
Guam
Malta
Portugal
France
Andorra
Norway
Bermuda
New Zealand
Cook Islands
United States Virgin Islands
Austria
Singapore
Australia
Puerto Rico
United States of America
Finland
Ireland
Canada
Iceland
Luxembourg
Sweden
Denmark
